# Stress Detection Using Experience Sampling: A Systematic Mapping Study

**DOI:** 10.3390/ijerph19095693

**Published:** 2022-05-07

**Authors:** Gulin Dogan, Fatma Patlar Akbulut, Cagatay Catal, Alok Mishra

**Affiliations:** 1 Department of Computer Engineering, Istanbul Kultur University, Istanbul 34158, Turkey; 1900000822@stu.iku.edu.tr (G.D.); f.patlar@iku.edu.tr (F.P.A.); 2 Department of Computer Science and Engineering, Qatar University, Doha 2713, Qatar; ccatal@qu.edu.qa; 3 Informatics and Digitalization Group, Faculty of Logistics, Molde University College-Specialized University in Logistics, 6410 Molde, Norway; 4 Software Engineering Department, Atilim University, Ankara 06830, Turkey

**Keywords:** systematic mapping study, experience sampling, emotion, stress detection, healthcare

## Abstract

Stress has been designated the “Health Epidemic of the 21st Century” by the World Health Organization and negatively affects the quality of individuals’ lives by detracting most body systems. In today’s world, different methods are used to track and measure various types of stress. Among these techniques, experience sampling is a unique method for studying everyday stress, which can affect employees’ performance and even their health by threatening them emotionally and physically. The main advantage of experience sampling is that evaluating instantaneous experiences causes less memory bias than traditional retroactive measures. Further, it allows the exploration of temporal relationships in subjective experiences. The objective of this paper is to structure, analyze, and characterize the state of the art of available literature in the field of surveillance of work stress via the experience sampling method. We used the formal research methodology of systematic mapping to conduct a breadth-first review. We found 358 papers between 2010 and 2021 that are classified with respect to focus, research type, and contribution type. The resulting research landscape summarizes the opportunities and challenges of utilizing the experience sampling method on stress detection for practitioners and academics.

## 1. Introduction

The chaos and rush of today make it impossible to live a stress-free life. People’s interactions with their colleagues and the environment in their daily lives create stress in varying degrees. Thus, stress is a factor and internal experience that causes changes in one’s life routine in response to leaving one’s comfort zone. Stress is an inner experience felt as a result of changes in a life routine in response to leaving one’s comfort zone. We can all feel stressed at work [1], with family [2], in traffic [3], and even with friends [4]. For example, at work, we may have to do a lot of work in a limited amount of time. Disagreements arising from differences of opinion between family members or friends can affect people negatively. As if all this were not enough, we may feel even more pressured in the face of external factors, such as the COVID-19 epidemic [5], wars [6], and economic crisis [7]. The human body still has primitive responses to stress as these reactions prepare the body for battle as if there is a real threat. The body adapts to the situation in various ways in order to survive. However, when we feel stressed all the time, this can lead to health problems as the body acts as if there is a constant threat [8]. For example, when we see a lion in real life, we feel a threat and our heart rate increases. However, the heart is a muscle, and just as our muscles swell when we do heavy sports, the heart can contract after a while when it works at a high tempo all the time. This situation is extremely dangerous for human health. Stress has physiological effects on people as well as psychological effects [9]. Effects such as depression [10], anxiety [11], and behavioral disorders [12] can be seen in individuals who are exposed to long-term stress.

According to the World Health Organization [13], stress is predicted to be one of the most important causes of dysfunction by 2020. It can threaten a person emotionally and physically, affecting their work performance and even health status [14]. Additionally, it is important to have appropriate emotional resilience and job satisfaction to increase work performance. According to Colligan and Higgins [15], stress in the workplace is detrimental to the well-being of employees and can lead to increased absenteeism, organizational dysfunction, and reduced productivity. Many studies have shown that attention interventions reliably reduce both general psychological stress [16,17,18] and occupational distress [19]. Furthermore, other evidence suggests that mindfulness is associated with coping success during stressful events [20].

Stress has a significant effect on every aspect of an individual’s life. The experience sampling approach emerges as one of the most effective ways to examine these effects. The experience sampling method (ESM), also known as ecological momentary assessment [21], is a structured daily technique that evaluates the current context and psychological events such as mood in daily life. In the experience sampling studies, participants are usually asked to answer various questions through a smartphone-based application. It is necessary to make instant self-evaluations from the participants who are notified by the mobile platform at various moments during the day. Thus, this method provides the opportunity to capture instant experiences in real-time. This method is used to collect data on different types of problems, such as addictive behavior [22,23,24], pain [25,26,27], evaluation of psychiatric problems [28,29], fatigue [30,31], and more recently, medical evaluations [32,33,34,35,36]. In particular, studies conducted in the working environment have been carried out to examine the mood of employees and investigate the positive and negative events that affect employees in the workplace.

This article presents the results of published studies on the practice of experience sampling that aim to shed light on trends and emerging practices in stress research. This study differs from previously published secondary papers in the literature [15,16] by presenting a thematic analysis instead of narrative summaries that focus on a qualitative review. The contributions of this study are three-fold:

- In this study, research articles on the experience sampling method and stress issues published between 2010 and 2021 were retrieved and evaluated;
- The issue of stress and experience sampling has not been systematically investigated until now, and to the best of our knowledge, this is the first review study using a systematic mapping approach for stress with experience sampling method;
- Selected primary studies have been evaluated from a wide variety of perspectives for identifying potential gaps in current research to identify areas for further investigation.

The remainder of the article is organized as follows. In the following section, the definition of the experience sampling method and its application principles is presented. In Section 3, the aims of the mapping study, research questions, and scope are defined. Following that, Section 4 describes how the systematic mapping methodology has been applied. The results of the mapping and the answers to the research questions are presented in Section 5. Section 6 compiles the results obtained from the research questions. The mapping of demographics, limitations, and future directions are presented in Section 6 and Section 7, respectively. Section 8 discusses the findings, and finally, Section 9 presents our conclusions.

## 2. Experience Sampling Approach

Experience sampling is one of the methods used to analyze emotional reactions [18] and habits. It aims to create a rich overview of the phenomena by examining the subject participating in the study, and the answers to a couple of questions are sought in the following process. One of the most critical methodological decisions in the study is to decide on question planning. The literature defines three different approaches for experience sampling methodology (ESM): random, time-based, and event-based [37]. As the name suggests, random sampling aims to collect data from the participants at non-periodic intervals. Random sampling is often preferred in scenarios where the indicators of the research topic cannot be determined. However, if the goal and focus of the research topic are specific, choosing other methods increases success. Time-based reporting requires participants to respond at specific times each day. In other words, it requires reports at the same time each day. This design is perfect for routine activities (such as how much food a participant eats) or easy-to-remember things. These exercises usually take about 1–2 weeks, as they do not require much time or effort. However, some researchers are concerned that such studies focus on a specific context. Consider a scenario in which test participants are viewed only at 9 o’clock while at work. Subject A appears to be more relaxed, while subject B displays higher stress levels. Thus, the participants may encounter a problem in a certain period of time, which can be considered the primary drawback of this approach. Event-based experience sampling studies require participants to answer questions about a specific event (e.g., smoking). These are extensively applied for social interactions, anger management, and stress detection. This design is the most reliable option for occasional special events. Since events are unpredictable, there is no need for specific schedule planning. With these methods, a mobile device is used during experience sampling. When the participants answer various objective questions through a program on a mobile device, they simultaneously create a ground truth with their smartphone instruments. Participants report their thoughts, feelings, behaviors, and/or surroundings at that moment or immediately after. The motivation of this process is to collect as much data as possible. More commonly used methods [38] to collect data include survey, interview, observation, document/record review, focus group, checklist, oral history, ethnography, case study, and experiment.

ESM has many benefits, but its implementation varies. The ecological validity of ESM is high because assessments are made in the natural course of real life [39]. While the metrics used in ESM design vary depending on the research question and the researcher’s preference, these metrics have various advantages and pitfalls in practice. One of the important factors is the correct equipment selection because the result is directly related to this choice. Certain decisions must be made between cost, effectiveness, practicality, and time spent. The most important limitation of the pen-and-paper method is the emergence of misleading data due to participants’ past or future tense responses. Technology-based designs, especially smartphone applications, increase data accuracy while providing more participants [40,41,42].

## 3. Goals, Questions, and Metrics

We used the Goal–Question–Metric (GQM) paradigm [43] to pose meaningful research questions in the evaluation of these systematic mapping goals, which are as follows:

- G1: To classify the articles of experience sampling applications regarding their application domain;
- G2: To understand the various perspectives of experience sampling (e.g., type of data, type of survey, coverage) that are investigated by the researcher;
- G3: To reveal the technologies and tools used for experience sampling;
- G4: To investigate how stress is triggered during the experience sampling and how the collected data is analyzed;
- G5: To analyze demographic and bibliometric data by identifying researchers and their affiliated organizations in this field;
- G6: To discover recent trends and future research directions in this area.

G1, G2, G3, and G4 are defined to reveal the practical use of the experience sampling method and understand the parameters that directly affect the participant–system interaction. These goals guide our initial research questions.

- RQ 1.1: What kind of data collection method was used in ESM?
- RQ 1.2: What type of data has been collected?
- RQ 1.3: How many participants were studied?
- RQ 1.4: How many questions were asked of the participants?
- RQ 1.5: What type of experience sampling method was used?
- RQ 1.6: What type of analysis method has been used?
- RQ 1.7: What type of stress was studied?
- RQ 1.8: What were the methods used to trigger stress?
- RQ 1.9: What was the average time spent on an experience sampling study?

To answer the questions listed, we examine the articles in detail, collect relevant metrics, create classifications that respond to the data and findings reported in the articles, and obtain frequencies. We do not provide a subjective view to answer any of these questions. Therefore, all measurements are objective. G1, G5, and G6 goals are about understanding the demographics and bibliometrics of articles and authors. These goals raise our second set of research questions listed below.

- RQ 2.1: Who are the authors with the most articles on experience sampling topics?
- RQ 2.2: Which countries produced the most articles?
- RQ 2.3: What is the academia/industry ratio of the author affiliations?
- RQ 2.4: Which venues have the highest number of articles?
- RQ 2.5: What is the annual publication trend?
- RQ 2.6: What are the most influential articles in terms of citation count?
- RQ 2.7: What is the number of citations by venue type?

The trends and limitations reported in the articles are extracted and presented to the readers. Question group 3 listed below serves this purpose.

- RQ 3.1: What limitations are reported in the papers?
- RQ 3.2: What lessons learned are reported?
- RQ 3.3: What future research directions are suggested?

**Figure 1:**
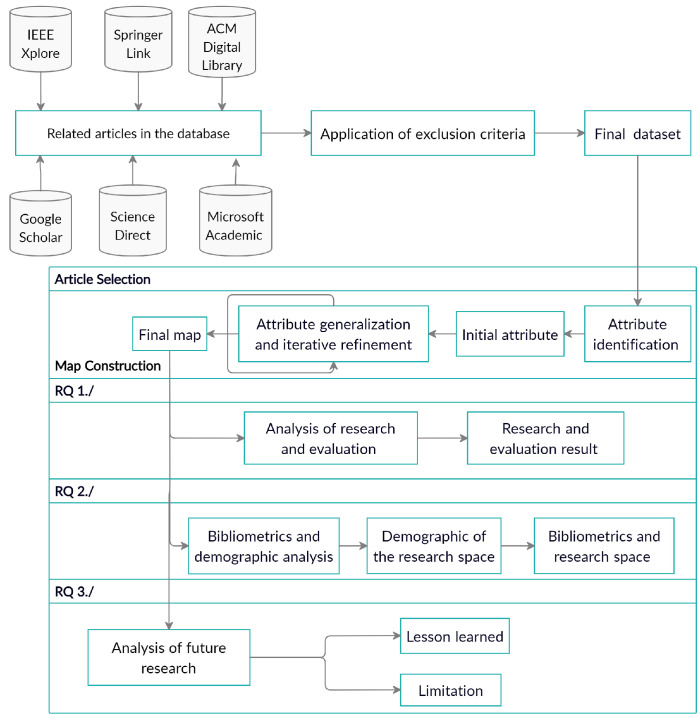
SM protocol. Article selection, Mapping, RQ 1, RQ 2, RQ 3.

The answers to the questions are based on the opinions and research results of the original authors who conducted the primary studies. After setting the study’s objectives, we linked them to the research questions and determined the metrics. The remainder of this study is based on the underlying protocol of this SM, as depicted in Figure 1, which describes the systematic mapping study’s workflow. We described the details in Section 4, Section 5, Section 6, Section 7 and Section 8.

## 4. Research Method

We chose to perform a systematic mapping study (SMS) to obtain an overview of the experience sampling method in stress detection and evaluation. We followed the guidelines provided in works, such as those of Kitchenham [44], Budgen et al. [45], and Petersen et al. [46].

### 4.1. Article Selection

The selection of the article actually forms the basis of the synthesis of all its conclusions. In this study, articles were selected using a three-step process, using the guidelines presented in the referenced systematic mapping article: (1) article identification using digital libraries and search engines, (2) exclusion criteria outside the scope of this study, and (3) definition and implementation of inclusion criteria targeting specific resources and locations that may be missed by digital libraries and search engines to manually select relevant articles. These steps are shown in Figure 1.

#### 4.1.1. Step 1: Article Identification

We acquired the literature list through a keyword-based search in electronic databases: IEEE Xplore, ACM Digital Library, Google Scholar, Microsoft Academic Search, Science Direct, and Springer Link. Search terms were selected through an emulated primary research term assessment process. The final results were converted into the following search terms: “Experience sampling*” and “Stress*.” In this step, 587 articles were obtained that made up the initial data pool.

#### 4.1.2. Step 2: Exclusion Criteria

Some eligibility criteria were established, and the following exclusion criteria were defined to eliminate articles from the initial repository.

- C1: Languages other than English;
- C2: Relevance to the topic;
- C3: Did not appear in the published proceedings of a journal, book, conference, symposium, magazine, or workshop.

The relevant criteria were applied sequentially. It was easier to apply criteria C1 and C2 than to apply criteria C3. Under the C3 criterion, each study was first reviewed by one author and then cross-checked by the other author to assess the relevance of the article. At the final stage of exclusion, 170 of the 587 articles were eliminated, and 417 articles remained for use in the study.

#### 4.1.3. Step 3: Final Article Set

**Figure 2:**
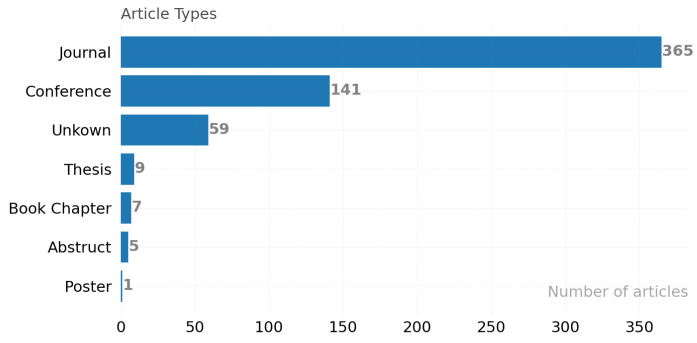
Distribution of article types.

Figure 2 presents the distribution of 417 articles analyzed during the study. Finally, a total of 358 articles (Appendix A) were used within the scope of the study. A few articles whose full texts were not available were classified as ”Others”.

### 4.2. Iterative Development of the Systematic Map

A systematic map is a tool used to classify selected articles. Map development is time-consuming because of the size of the task and the complex process. Our map, which contains 358 article reviews, employs the GQM approach, which contains research questions and metrics used as the primary guide to the SMS. For RQ 1, we need to collect the following attributes: “data collection method,” “data type,” “number of participants,” “number of questions,” “experience sampling method,” “analysis method,” “stress type,” “methods to trigger stress,” and “mean time.” We defined these attributes and presented the map structure that can be identified as comprehensive. Similarly, for RQ 2, we need to collect the following metrics: “locations with the highest number of articles,” “authors with maximum articles,” “author memberships,” “number of articles per year,” “number of articles by venue type,” “number of citations by venue type,” and “number of citations by location.” This guide is used to reveal objective demographic and bibliometric data for the authors and articles.

Conclusively, we need to obtain the following metrics for RQ 3: “limitations,” “lessons learned,” and “future research directions.” This directed us to create our third map, which establishes the basis for clarifying RQ 3.

## 5. Mapping Research and Evaluation

**RQ 1.1:** What kind of data collection method was used in ESM?

**Figure 3:**
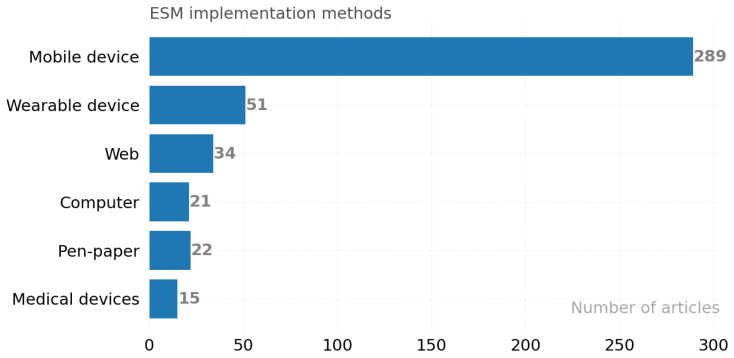
Platforms/Devices used to collect data in articles.

Within the framework of experience sampling, demographic information and instant experiences can be collected via traditional paper-based methods, web-based methods [47,48], mobile devices-based such as mobile phone, personal digital assistant (PDA), wearable devices such as Siemens 3T scanner, Holter monitor, Empatica e4, Real Extraction DNA kit, Affectiva Q-Sensor, HealthPatch MD, Google Glass, Emotiv Insight EEG Headset, Microsoft Band 2, WatchMinder3, Philips Respironics [49], and/or medical devices such as a magnetic resonance (MR) device [50]. Figure 3 shows the number of articles in these categories. Each article is associated with at least one or more categories, for example, Ref. [51] collecting voice and questionnaire data with a mobile device while simultaneously collecting physiological signals with an MR device.

As shown in Figure 3, the most popular ES method used by 289 of the researchers was the mobile device. The second most widely used method was wearable devices (51), which provided new data sources for research.

**RQ 1.2:** What Type of Data Has Been Collected?

**Figure 4:**
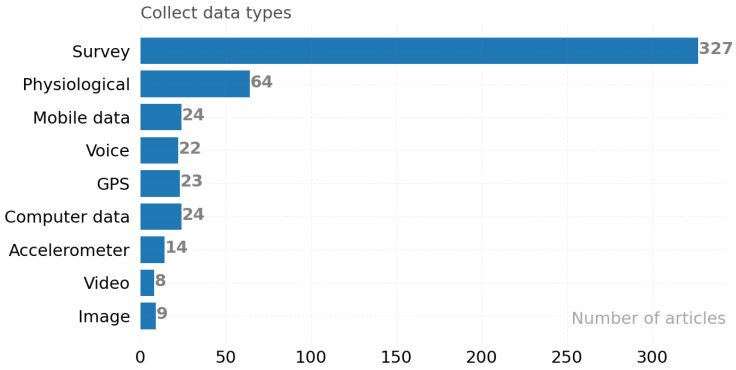
Types of data collected in articles.

**Figure 5:**
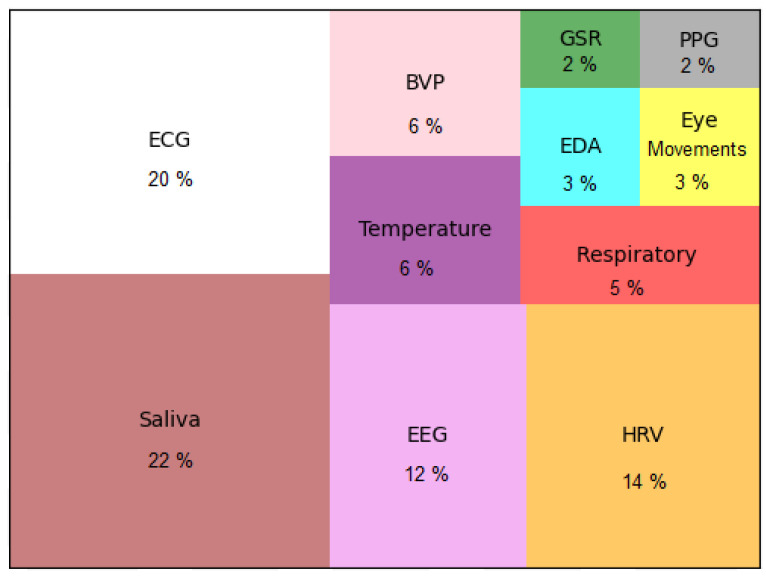
Types of physiological data collected in articles.

In Figure 4, all data collection methods used within the scope of 358 articles are divided into the following main categories: survey, physiological data, mobile data, audio, GPS, accelerometer, video, image, and computer-generated data. The data types collected in the researched articles have provided various data such as heart rate, skin temperature, and pulse using wearable devices [52,53]. We describe such data within the context of physiological data and show it in Figure 5. Mobile data and PC data track daily metrics of the type of frequency and clicks in stressful situations. GPS is used to obtain location information of a person in a stressful situation to search for a link between stress and geographic location. Physiological data are divided into the following categories: saliva, electrocardiography (ECG), electroencephalography (EEG), heart rate variability (HRV), temperature, blood volume pulse (BVP), respiration, eye movements, electrodermal activity (EDA), galvanic skin response (GSR), and photoplethysmography (PPG). The x-axes in Figure 4 show the number of articles and y-axis data types for each data type collected.

**Figure 6:**
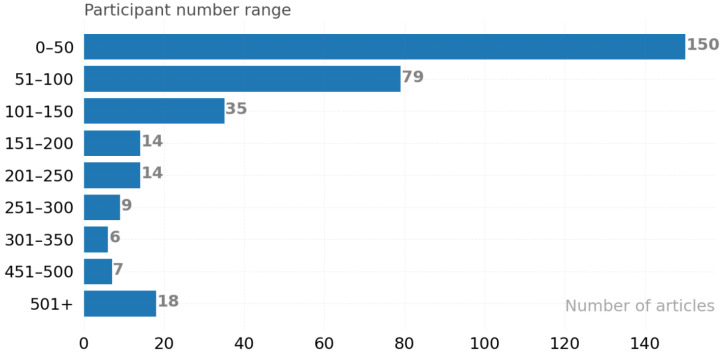
Distribution of the number of participants.

**RQ 1.3:** How many participants were studied? Within the scope of the articles examined in Figure 6, data from 82,798 participants were analyzed. The x-axis of the graph shows the number of articles, and the y-axis shows the participant range according to the number of participants in the study. The most preferred participant number range (150 articles) was detected as 0–50 participants. Two common reasons for conducting studies with fewer participants were found: one is the participants’ personal data security [54], and the other is the work in specific areas [55].

**Figure 7:**
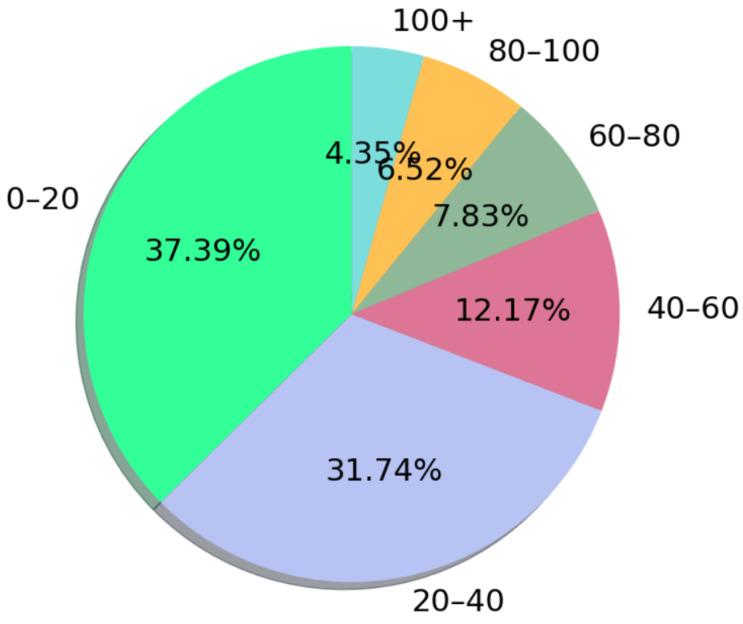
Distribution of the questions used according to the intervals.

**RQ 1.4:** How many questions were asked of the participants? A total of 6998 questions were identified within the reviewed studies’ scope, as shown in Figure 7. As seen in the graph, the most preferred (38%) question range was determined as 0–20. It is emphasized that keeping the question texts short to not distract the participant’s attention ensures more correct answers and ease of implementation [S059].

**RQ 1.5:** What experience sampling method was used?

**Figure 8:**
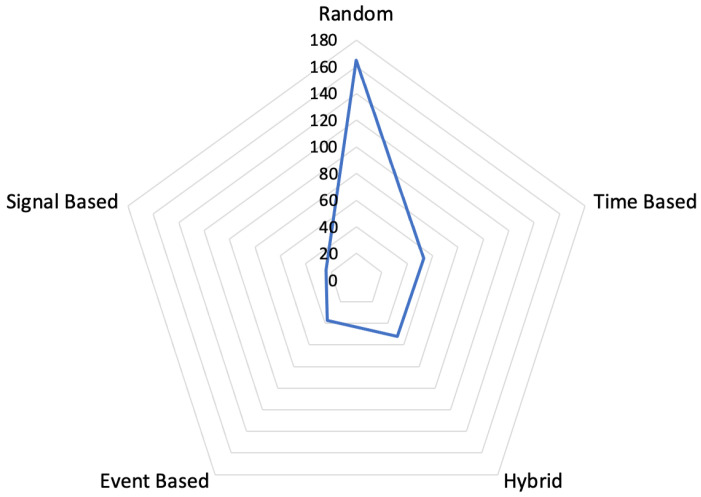
Types of the experience sampling method.

Types of ESM used in the articles are given in Figure 8. Random ESM [56] is used to sample the participant’s experience at unforeseen times. Time-based ESM [57] sends survey notifications at certain hours within the participant’s information. A semi-random, half-time-based ESM [58], random survey notification is made in a certain time frame within the participant’s information. The event-based method [59] is applied before and after certain actions are performed by the participants. In the signal-based method [S152,S288] a signal is sent to the participant to answer the questionnaire at unpredictable times. Each article is associated with at least one or more categories. For example, in [60], experience sampling data were collected from participants for four days based on the signal, four days based on the event, and four days based on time.

**RQ 1.6:** What analysis method was used?

**Figure 9:**
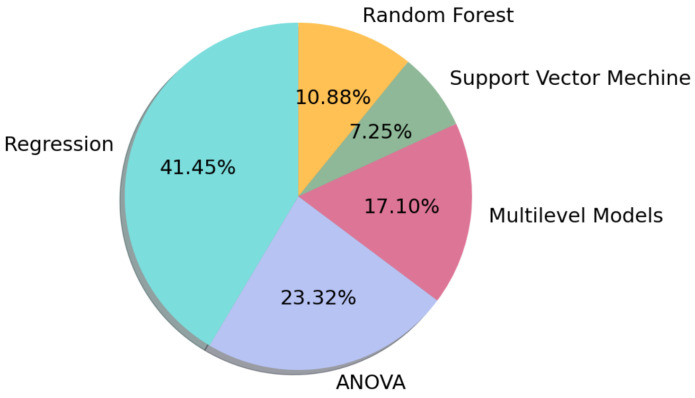
Analysis methods used in the primary studies.

We categorized the data analysis methods used in primary studies as regression, analysis of variance (ANOVA), multi-level models, random forest, and support vector machine. To rank data analysis methods based on the number of methods used in the published articles, the methods were extracted (Figure 9). The most popular method used for data analysis, as shown in Figure 9, is regression methods (41%). The second most popular method is ANOVA (23%), and the third is the statistical multi-level model (41%), also known as a random parameter model that contains hierarchical linear models, linear mixed-effect models, mixed models, nested data models, random coefficient, and random-effects models (17%).

**RQ 1.7:** What type of stress was studied?

**Figure 10:**
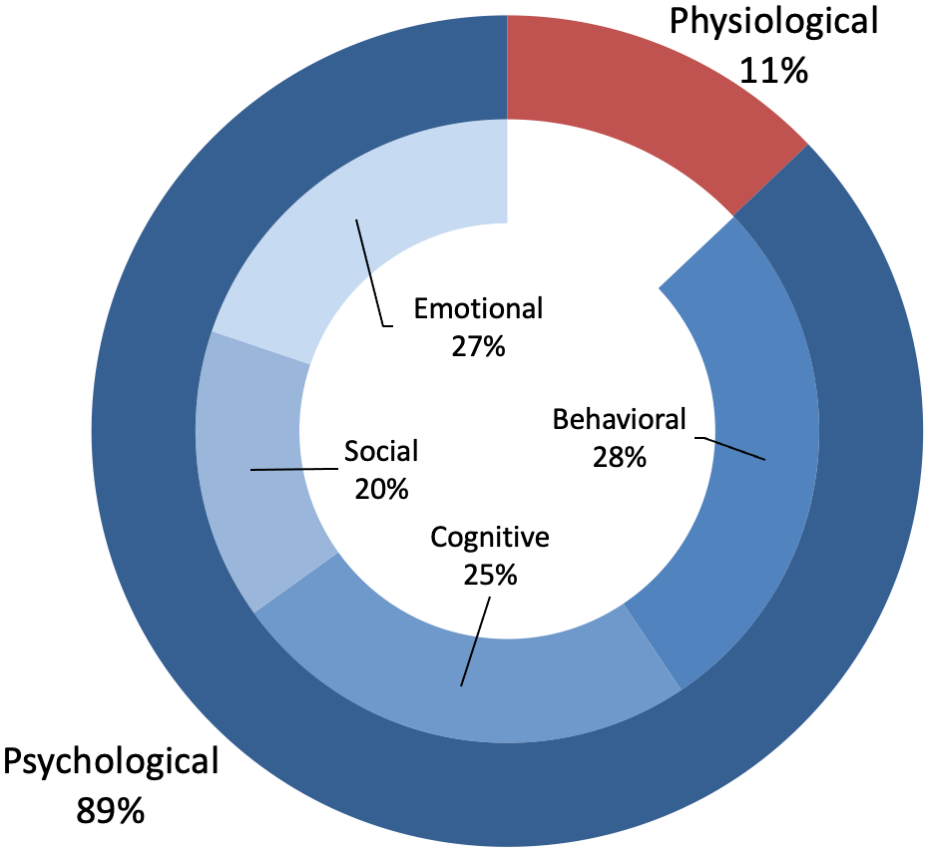
Distributions of physiological and psychological stress studies.

Stress occurs when the body is affected by a number of negative events and shows physiological and psychological reactions to them. While physiological stress affects human physiology, psychological stress can affect social life, movement/behavior, cognitive, and emotional areas. In Figure 10, the physiological and psychological stress types of the participants were compared. Most participants in the studies (89%) felt psychological stress, and 11% felt physiological stress. The distribution of the psychological stress types on the participants can be displayed. While the most common type of psychological stress felt was behavioral (28%), the second was cognitive (25%), then social (20%) and emotional (27%) stress.

**RQ 1.8:** What specific focus was established on triggering events of stress?

**Figure 11:**
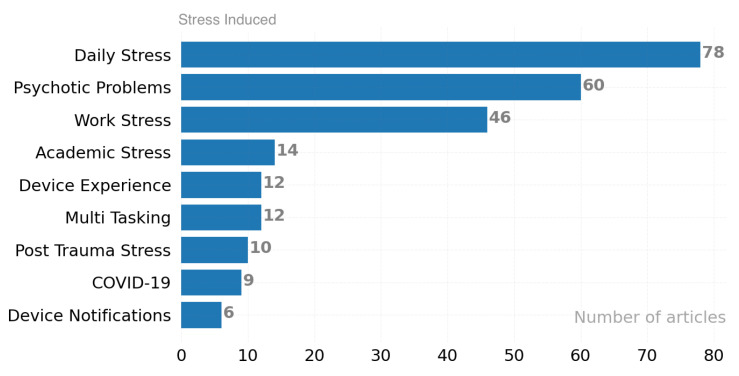
Triggering events that have been revealed.

The ten factors that most frequently cause stress in the studies reviewed are shown in Figure 11. The number of x-axis runs is triggering events that cause y-axis stress. Most of the studies aimed at coping with daily stress (78) and improving the current situation, while other important stress causes can be evaluated as psychotic problems (60) and work stress (46).

**RQ 1.9:** What was the average time spent on an experience sampling study?

**Figure 12:**
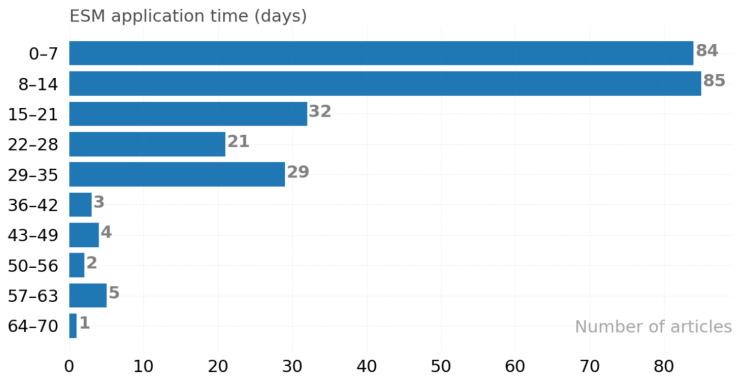
Time spent sampling experience.

The user experience data collection time is shown in Figure 12 with certain days; the x-axis reflects the number of studies, and the y-axis is the number of user experience collection days. The most preferred data collection period is 8 to 14 days. Some studies may require longer observations than others due to the subject matter. For example, [S227] is a study with an observation of three months or more.

**Figure 13:**
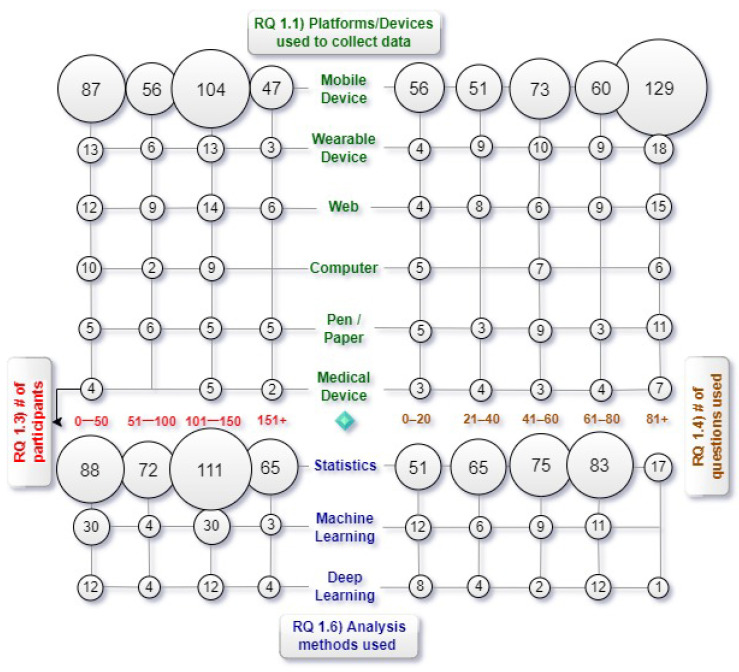
Mapping results obtained from the combination of Q1, Q3, Q4, and Q6. (Note that some studies may serve on multiple perspectives, e.g., studies with multiple platforms occupied S054, S062, and S084, and multiple analysis methods used S162 and S352).

Figure 13 shows the mapping results obtained from research sub-questions Q1.1 (Data Collection Method) and Q1.3 (Number of Participants) in comparison to research sub-questions Q1.4 (Number of Questions) and Q1.6 (Analysis Method). These results may indicate that:

- Most ESM studies are designed to target a maximum of 100 participants in the experiment, and mobile devices are the most preferred data collection method, among others, especially in cases where the number of participants increased, where the use of mobile devices was considered almost the only option;
- The number of questions asked of the participants was mostly limited to 40. Similarly, the mobile device appears to be the method that supports the greatest number of questions;
- Examination of the analysis method according to the number of participants shows that there is no pattern. In any case, statistical analysis is the most widely adopted technique.

**Figure 14:**
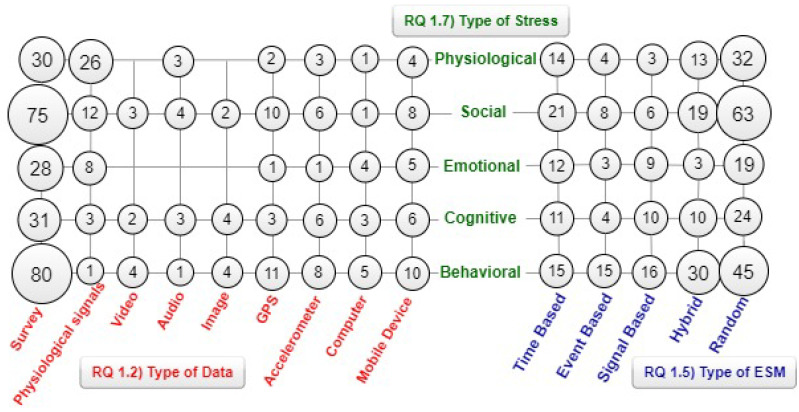
Mapping results obtained from the combination of Q2, Q5, and Q7.

Figure 14 shows the mapping results obtained from research sub-question Q1.7 (Research Focus) in comparison to research sub-questions Q1.2 (Data Type) and Q1.5 (ESM Approach). These results may indicate that:

- The most used ESM approach is random sampling, even when the research focus changes. This approach, which dominates the studies in the analysis of physical stress, is used in conjunction with other approaches in the analysis of mental stress;
- While the most used data collection method for physical stress is the acquisition of physiological signals, the other research focus is surveys.

## 6. Mapping Demographics

In this section, we address RQ 2 and examine the demographics of articles and authors.

RQ 2.1: Who are the authors with the most articles on experience sampling topics?

Inez Myin-Germeys is the most published and the most senior author, with 18 articles in this field. The second- and third-ranked authors were Jim Van Os and Marieke Wichers, with 15 and 10 articles, respectively.

RQ 2.2: Which countries produced the most articles?

The places that produced the most work in the field of stress and experience sampling were examined. As a result of this review, USA (84) ranks first among the places that conduct the most research in this field. The UK (20) is in second place, and Netherlands (15) is third.

RQ 2.3: What is the academia/industry ratio of the author affiliations?

It was investigated how much the academia and industry contributed to the studies in the field of stress and experience sampling. The Academy has made a great contribution to the literature with 173 studies. However, it has been observed that the studies in this field are not yet mature in the sector.

RQ 2.4: Which venues have the highest number of articles?

**Figure 15:**
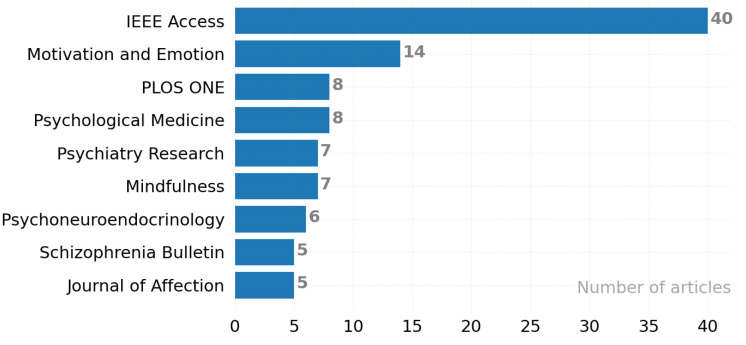
The ten most preferred journal titles.

Figure 15 aims to present the top journals in the field because our main motivation is to raise awareness about which resources researchers should follow. Most preferred is IEEE Acsess (40). Motivation and Emotion (14) ranked second, and Psychological Medicen and PLOS ONE (8) ranked third.

RQ 2.5: What is the annual publication trend?

Table 1 shows the distribution over time for the 358 primary studies. With 50 articles published since 2010, it has been determined that the most active year is 2019.

RQ 2.6: What are the most influential articles in terms of citation count?

When the top three articles with the most citations are examined, the first place belongs to [S237] with 355 citations, and the second and third ones are [S140] and [S313], with 328 and 265 citations, respectively.

RQ 2.7: What is the number of citations by venue type?

**Figure 16:**
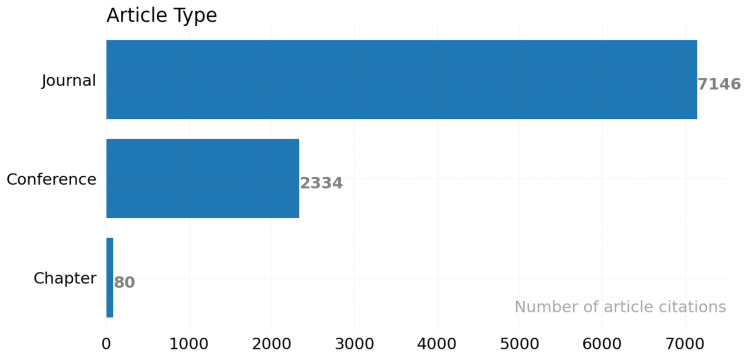
Article classification by venue type.

According to the venue type, we classify the articles into three categories: conference, journal, and book. In Figure 16, the x-axis shows the number of articles in each category, and the y-axis shows the venue type. Figure 16 shows places with a high number of journals (260). When the number of citations and type of place is collected for each article, we observed that journal articles receive the most citations, with 7146 citations.

## 7. Mapping Limitations and Future Directions

Evidence from the reviewed primary studies indicates that applying EMS can be challenging from different perspectives. The research questions RQ 3 attempted to identify existing limitations and useful ESM approaches that can provide direction for future studies.

RQ 3.1: What limitations are reported in papers?

Limitations of the published studies are broadly categorized based on the participants, size of the dataset, analysis technique, algorithm, device/tool used, scaling, applicability, and causality perspectives.

- Participants: When the test is limited to a small group of participants, includes participants of similar socioeconomic status, only healthy individuals, only women or only men, or certain groups of participants, such as certain workgroups, generalization cannot be made [S002]. The need for a dataset to include all possible representative characteristics in a balanced structure may not be met in real life.
- Size of the Dataset: If the sample size is too small or large, it reduces the power of the work; for example, a large data size causes difficulties in processing data [S050]. On the other hand, lack of data negatively affects the accuracy of analysis models [S063]. Note that the use of simple classifiers such as Naive Bayes is recommended when working with a small dataset [S066]. As an alternative method, a large dataset belonging to the same or a close domain can be used as a reference with the transfer learning approach. Data augmentation, on the other hand, aims to synthetically reproduce existing data as the last proposed technique.
- Analysis technique: Possibly misleading situations may be encountered during the analysis, e.g., noisy data, feature extraction error, misleading data from participants, which will cause the analysis to fail [61].
- Algorithm: Known limitations of the algorithms presented, for example, Akaike information criterion (AIC) gives information about the quality of the model in an absolute sense. It will not give any warning if all candidate models are bad [S034].
- Device: Device and tool limitations, such as data accuracy and adequacy problems, can occur due to device difficulties such as battery power, consumption of a computational resource, and difficulty of the calibration [S177].
- Tool: Limitations of developed software, for example, collecting missing data due to software deficiencies, will result in incomplete deductions [S239].
- Scaling: The limitations of determining the methods to be used, for example, insufficient data collection time, cause the data size not to reach the optimum level [S241].
- Applicability: Limitations on usability under different environments, for example, the fact that its use appeals to an overly specific audience, cause it not to be preferred by the rest of the users [62].
- Causality: Objectivity, the limitation of unprovable, for example, subjective or misleading responses from participants, makes the study ungeneralizable [S072].

**Figure 17:**
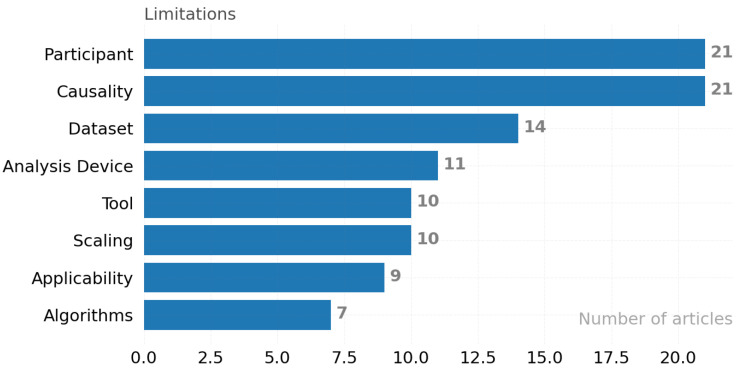
Distribution of the reported limitations by categories.

In this SM, the limitations are objectively stated as specified by each study author. Out of 358 articles, 103 articles reported one or more limitations of the research study. The obtained limitations are shown in Figure 17. The figure depicts the limitations of the research. The x-axis displays the number of articles in each category, and the y-axis recites categories. The most common limitation is the participant category.

RQ 3.2: What lessons learned are reported?

Most of the authors reported lessons learned from their studies. The lessons learned were reported in 81.84% (293/358) of the articles. Lessons learned vary greatly depending on the individual research and study context. That is why we conducted a qualitative analysis rather than a quantitative analysis. Readers should note that it must be interpreted in the context of the studies.

Apart from participant questionnaires, supportive data can be obtained with various devices such as Likert-scale, open-ended, or visual questions. More detailed studies were conducted with MR, Holter, or mobile device data to contribute to the literature [62,63]. Among these supporting data, photographs can be annotated with more than one tag, depending on the context. Following the participants longer and asking them to rate their mood on the photos provided more evidence for the temporal effect of happiness from different sources [S054].

Stress problems cause a decrease in employees’ performance at work [64], a decrease in the academic achievement of students at school [65], depression in healthy individuals in daily life [66], and diseases in individuals with genetic diseases [67]. It has been determined that individuals who have experienced childhood trauma [68] or war trauma [69] were more sensitive to stress. Due to the increasing stress in society, web [70] and mobile [71] health applications have been developed, and awareness studies have made it possible to cope with stress better [72].

RQ 3.3: What future research directions are being suggested? Most of the articles provided guiding advice for ongoing research. These can be broadly divided into the following categories:

- Participant: Participant-based improvements—Designing a study where participants can give direction to the study increases overall performance [S189].
- Dataset: Develop methods for collecting participant data, such as collecting large datasets [S082]. Big dataset analysis with a balanced structure always results in more meaningful inferences.
- Analysis: We observed that statistical methods are the most preferred in the analysis of the collected data. In the last quarter of the decade researched, there is a tendency to use more machine learning and deep learning approaches. Especially with the adoption of cloud computing and GPU-based processing technologies, analysis processes can be accelerated [S050].
- Algorithm: Build new models with different algorithmic approaches, such as semi-supervised deep learning approaches [S058] using ensemble models.
- Model: The goal is to improve the protocols used in the studies, such as a universal background model [S035].
- Various Context Indexes: Using data from multiple devices depending on the context, such as wearable device data [S147]. Models fed with data collected from different dimensions and perspectives have higher performance.
- Scaling: Scales used across the study, such as data collection time [S082]. Mostly, the time scale is used by default in studies. The contribution of analyses to be made with different scales should be investigated.
- Applicability: Developing useful and accurate tools, such as an application developed for individuals with severe cognitive impairment [S010]. Most studies use commodity systems to collect data and off-the-shelf business intelligence (BI) tools for analysis. Some situations do not accept these standard approaches. Software engineering approaches should be leveraged for problem-tailored tooling.

**Figure 18:**
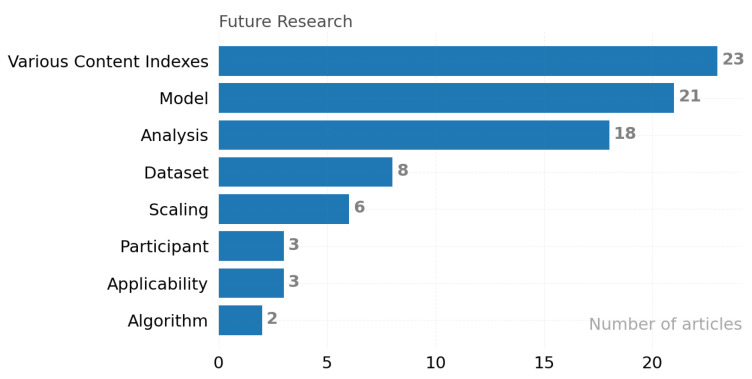
Future research directions.

Future research directions outlined in the articles were identified. Figure 18 exhibits these data. Although these data contain directions for future research, they help us understand the thoughts of the researchers and what they perceived as missing parts at the time of their studies and publication.

In Figure 18, the x-axis shows the number of articles in each category, and the y-axis displays categories. Various context indexes, multi-device ESM work (23 articles) is perceived as the area that requires the most study. Model (21 articles) is perceived as the second area that requires the most study.

## 8. Discussion

This section summarizes the main findings of this systematic mapping study detailed in previous chapters. In addition, it highlights limitations that may represent threats to its validity and discusses implications for research and practice.

### 8.1. Principal Findings

The results provide an objective summary of trends in the stress-based experience sampling method. The collected data shows that context-dependent indexes, models, and analysis methods attract significant attention in the research community (Figure 18). The collection of new data types provided by the developing sensor technology, modeling with new method combinations, and obtaining meaningful information from these data due to various analyses was carried out by [S133] in the laboratory environment. However, real-life usability tests (Figure 18) are still ongoing. Psychological behavioral stress is the most researched topic for coping with depression, anxiety, panic disorders, and anger issues, which are some of the biggest problems of our time. Studies under this category aimed to identify the self-destructive or unhealthy behaviors of participants. These studies were considered to have a high potential for productization.

### 8.2. Limitations of the Systematic Mapping Study

The conducted systematic mapping study suffers from several limitations. The principal limitations are identified as selection bias, inaccuracy in data extraction, and misclassification. Selection bias refers to the distortion of a statistical analysis caused by the criteria used to select publications. To avoid this limitation, we aimed to determine the ideal query string for the selection of papers and include multiple repository searches, as explained in Section 4.

Inaccuracy and misclassification in data extraction refers to the possibility that information from a primary study and the information extracted will be interpreted differently by reviewers. To alleviate this threat, both authors independently reviewed all articles, and any discrepancies that emerged were resolved by consensus. We aimed to address different aspects of this research field by using several research questions; however, covering all aspects and the contributing factors is nearly impossible. Different researchers can consider addressing other aspects that we did not include in this research.

### 8.3. Implications for Research and Practice

The findings of our systematic mapping study have implications for researchers planning new studies of ESM on stress and for practitioners designing new products for the ecosystem. The most critical challenge is the absence of a product designed for use in stress analysis. Another important finding of our secondary study is that the papers in which statistical analysis and machine learning models were combined reached more comprehensive results with high accuracy. Therefore, we recommend that researchers apply accurate statistical data analysis, methods, and techniques that follow state-of-the-art machine learning approaches, especially deep learning.

Our findings show that the majority of the papers reported that participants are less motivated to engage in long-term experiments. In particular, the ergonomics of the mobile device/sensor system used allow more questions to be asked or more data to be collected. We, therefore, consider that there is an important shortage of dedicated equipment for such research projects. From a management perspective, designing a non-invasive wearable system that can be used without affecting daily life will be an effective solution to this limitation.

## 9. Conclusions

The aim of this study was to understand the stress-based experience sampling applications through a systematic mapping study of 358 research articles from well-known repositories. We aimed to explore the current major trends in the research area and how different ESM approaches are studied. As a result of the investigations on ESM and stress, we observed that although the cooperation between academia and industry is increasing, the industry has not had much one-to-one effect on research projects. With the widespread use of sensor technology, data collection has become easier, and its application has become practical. Secondly, we found that physical stress was less studied, with the majority of studies focusing on mental stress, especially cognitive and behavioral aspects. We also observed that random and hybrid sampling from ESM methods are preferred, and deep learning approaches have come to the fore in the establishment of the analysis model in recent years. The main reason the observation periods of the studies were mostly up to two weeks was understood as the limitations of the equipment used and the participants’ unwillingness to participate in long-term experiments. Finally, our aim for bibliometric analysis is to direct the researchers in this field to different studies that provide useful information. For instance, popular venues for publication, country-wise analysis, most cited research papers, and most active researchers are reported in this study. Despite the great emphasis placed on stress in the literature, none of the commercially available tools are geared toward stress-based remediation. The software of such systems needs to be tuned for a suitable use for stress analysis. Our survey revealed that the researcher who has performed the most work in this field has 18 publications and has contributed to the most cited work. These researchers generally conduct their research in the USA, and the UK has also been observed. This means that researchers who want to take part in studies in this field can take part in the study groups in the USA and UK, where the most studies in this field are performed. It is seen that the most preferred journal is the IEEE Access journal, which has been chosen 40 times by far.

## Figures and Tables

**Table 1:** Distributions over publication types.

Type of Publication	2010	2011	2012	2013	2014	2015	2016	2017	2018	2019	2020	2021	Total	
Conference		4	2	5	14	10	8	20	9	16	3	2	93	25.97%
Journal	10	8	14	15	16	16	22	25	39	34	23	37	259	72.34%
Book Chapter				1	1		1	1	1			1	6	1.67%
Total	10	12	16	21	31	26	31	46	49	50	26	40	358	100%

## Data Availability

Not applicable.

## Appendix A. Primary Studies Selected

[S001] Weppner, J., Lukowicz, P., Serino, S., Cipresso, P., Gaggioli, A., and Riva, G. (2013, May). Smartphone based experience sampling of stress-related events. In 2013 7th International Conference on Pervasive Computing Technologies for Healthcare and Workshops (pp. 464–467). IEEE.
[S002] Gomes, P., Kaiseler, M., Queirós, C., Oliveira, M., Lopes, B., and Coimbra, M. (2012, January). Vital Analysis: Annotating sensed physiological signals with the stress levels of first responders in action. In 2012 Annual International Conference of the IEEE Engineering in Medicine and Biology Society (pp. 6695–6698). IEEE.
[S003] Sano, A., Johns, P., and Czerwinski, M. (2017, October). Designing opportune stress intervention delivery timing using multi-modal data. In 2017 Seventh International Conference on Affective Computing and Intelligent Interaction (ACII) (pp. 346–353). IEEE.
[S004] Lutchyn, Y., Johns, P., Czerwinski, M., Iqbal, S., Mark, G., and Sano, A. (2015, September). Stress is in the eye of the beholder. In 2015 International Conference on Affective Computing and Intelligent Interaction (ACII) (pp. 119–124). IEEE.
[S005] Gomes, P., Kaiseler, M., Lopes, B., Faria, S., Queirós, C., and Coimbra, M. (2013, July). Are standard heart rate variability measures associated with the self-perception of stress of firefighters in action?. In 2013 35th Annual International Conference of the IEEE Engineering in Medicine and Biology Society (EMBC) (pp. 2571–2574). IEEE.
[S006] Ciman, M., and Wac, K. (2016). Individuals’ stress assessment using human-smartphone interaction analysis. IEEE Transactions on Affective Computing, 9(1), 51–65.
[S007] Smyth, J. M., and Heron, K. E. (2016, October). Is providing mobile interventions “just-in-time” helpful? An experimental proof of concept study of just-in-time intervention for stress management. In 2016 IEEE Wireless Health (WH) (pp. 1–7). IEEE.
[S008] Ciman, M., Wac, K., and Gaggi, O. (2015, May). iSenseStress: Assessing stress through human-smartphone interaction analysis. In 2015 9th International conference on pervasive computing technologies for healthcare (PervasiveHealth) (pp. 84–91). IEEE.
[S009] Ghosh, S., Ganguly, N., Mitra, B., and De, P. (2017, January). Towards designing an intelligent experience sampling method for emotion detection. In 2017 14th IEEE Annual Consumer Communications and Networking Conference (CCNC) (pp. 401–406). IEEE.
[S010] Wohlfahrt-Laymann, J., Hermens, H., Villalonga, C., Vollenbroek-Hutten, M., and Banos, O. (2018, March). Enabling remote assessment of cognitive behaviour through mobile experience sampling. In 2018 IEEE International Conference on Pervasive Computing and Communications Workshops (PerCom Workshops) (pp. 794–799). IEEE.
[S011] Paredes, P., Sun, D., and Canny, J. (2013, May). Sensor-less sensing for affective computing and stress management technology. In 2013 7th International Conference on Pervasive Computing Technologies for Healthcare and Workshops (pp. 459–463). IEEE.
[S012] Van Calster, L., D’Argembeau, A., Salmon, E., Peters, F., and Majerus, S. (2017). Fluctuations of attentional networks and default mode network during the resting state reflect variations in cognitive states: evidence from a novel resting-state experience sampling method. Journal of Cognitive Neuroscience, 29(1), 95–113.
[S013] Ghandeharioun, A., McDuff, D., Czerwinski, M., and Rowan, K. (2019, September). Towards understanding emotional intelligence for behavior change chatbots. In 2019 8th International Conference on Affective Computing and Intelligent Interaction (ACII) (pp. 8–14). IEEE.
[S014] Kuutila, M., Mäntylä, M. V., Claes, M., and Elovainio, M. (2018, June). Daily questionnaire to assess self-reported well-being during a software development project. In Proceedings of the 3rd International Workshop on Emotion Awareness in Software Engineering (pp. 39–43).
[S015] Alibasa, M. J., Calvo, R. A., and Yacef, K. (2019). Sequential pattern mining suggests wellbeing supportive behaviors. IEEE Access, 7, 130133–130143.
[S016] Kaji, H., Iizuka, H., and Sugiyama, M. (2018). ECG-based concentration recognition with multi-task regression. IEEE Transactions on Biomedical Engineering, 66(1), 101–110.
[S017] Vhaduri, S., and Poellabauer, C. (2016, October). Human factors in the design of longitudinal smartphone-based wellness surveys. In 2016 IEEE International Conference on Healthcare Informatics (ICHI) (pp. 156–167). IEEE.
[S018] Alibasa, M. J., and Calvo, R. A. (2019, September). Supporting mood introspection from digital footprints. In 2019 8th International Conference on Affective Computing and Intelligent Interaction (ACII) (pp. 96–101). IEEE.
[S019] Pryss, R., Schlee, W., Langguth, B., and Reichert, M. (2017, June). Mobile crowdsensing services for tinnitus assessment and patient feedback. In 2017 IEEE International Conference on AI and Mobile Services (AIMS) (pp. 22–29). IEEE.
[S020] Ghandeharioun, A., McDuff, D., Czerwinski, M., and Rowan, K. (2019, September). EMMA: an emotion-aware wellbeing chatbot. In 2019 8th International Conference on Affective Computing and Intelligent Interaction (ACII) (pp. 1–7). IEEE.
[S021] Meyer, A. N., Satterfield, C., Züger, M., Kevic, K., Murphy, G. C., Zimmermann, T., and Fritz, T. (2020). Detecting Developers’ Task Switches and Types. IEEE Transactions on Software Engineering.
[S022] Ghosh, S., Sahu, S., Ganguly, N., Mitra, B., and De, P. (2019, January). EmoKey: An emotion-aware smartphone keyboard for mental health monitoring. In 2019 11th International Conference on Communication Systems and Networks (COMSNETS) (pp. 496–499). IEEE.
[S023] Cheng, S. Y., Wu, X., and Liou, S. (2016, December). Health training APP design: A comprehensive model of mindfulness feedback system. In 2016 International Conference on Orange Technologies (ICOT) (pp. 88–91). IEEE.
[S024] Pradhan, S., Qiu, L., Parate, A., and Kim, K. H. (2017, May). Understanding and managing notifications. In IEEE INFOCOM 2017-IEEE Conference on Computer Communications (pp. 1–9). IEEE.
[S025] Ghosh, S., Goenka, S., Ganguly, N., Mitra, B., and De, P. (2019, September). Representation Learning for Emotion Recognition from Smartphone Keyboard Interactions. In 2019 8th International Conference on Affective Computing and Intelligent Interaction (ACII) (pp. 704–710). IEEE.
[S026] Ghosh, S., Ganguly, N., Mitra, B., and De, P. (2017, October). Evaluating effectiveness of smartphone typing as an indicator of user emotion. In 2017 Seventh International Conference on Affective Computing and Intelligent Interaction (ACII) (pp. 146–151). IEEE.
[S027] Seeling, P. (2015, June). Augmented vision and quality of experience assessment: Towards a unified evaluation framework. In 2015 IEEE International Conference on Communication Workshop (ICCW) (pp. 1735–1740). IEEE.
[S028] Bota, P. J., Wang, C., Fred, A. L., and Da Silva, H. P. (2019). A review, current challenges, and future possibilities on emotion recognition using machine learning and physiological signals. IEEE Access, 7, 140990–141020.
[S029] Sarkar, S., and Parnin, C. (2017, May). Characterizing and predicting mental fatigue during programming tasks. In 2017 IEEE/ACM 2nd International Workshop on Emotion Awareness in Software Engineering Emotion) (pp. 32–37). IEEE.
[S030] Wac, K., and Tsiourti, C. (2014). Ambulatory assessment of affect: Survey of sensor systems for monitoring of autonomic nervous systems activation in emotion. IEEE Transactions on Affective Computing, 5(3), 251–272.
[S031] Ickin, S., Wac, K., and Fiedler, M. (2013, November). Qoe-based energy reduction by controlling the 3g cellular data traffic on the smartphone. In 2013 22nd ITC specialist seminar on energy efficient and green networking SEEGN) (pp. 13–18). IEEE.
[S032] Handte, M., Foell, S., Wagner, S., Kortuem, G., and Marrón, P. J. (2016). An internet-of-things enabled connected navigation system for urban bus riders. IEEE internet of things journal, 3(5), 735–744.
[S033] Elton, A., and Gao, W. (2015). Task-positive functional connectivity of the default mode network transcends task domain. Journal of Cognitive Neuroscience, 27(12), 2369–2381.
[S034] Emerencia, A. C., Van Der Krieke, L., Bos, E. H., De Jonge, P., Petkov, N., and Aiello, M. (2015). Automating vector autoregression on electronic patient diary data. IEEE Journal of Biomedical and Health Informatics, 20(2), 631–643.
[S035] Deshpande, G., Viraraghavan, V. S., Duggirala, M., Reddy, V. R., and Patel, S. (2017, October). Empirical evaluation of emotion classification accuracy for non-acted speech. In 2017 IEEE 19th International Workshop on Multimedia Signal Processing (MMSP) (pp. 1–6). IEEE.
[S036] Gustarini, M., Marchanoff, J., Fanourakis, M., Tsiourti, C., and Wac, K. (2014, October). UnCrowdTPG: Assuring the experience of public transportation users. In 2014 IEEE 10th International Conference on Wireless and Mobile Computing, Networking and Communications (WiMob) (pp. 1–7). IEEE.
[S037] Theilig, M. M., Korbel, J. J., Mayer, G., Hoffmann, C., and Zarnekow, R. (2019). Employing environmental data and machine learning to improve mobile health receptivity. IEEE Access, 7, 179823–179841.
[S038] Fritzsche, E. S., Schlingensiepen, J., and Kordts-Freudinger, R. (2018, April). Study motivation and academic emotions in engineering students: A case study in german higher education. In 2018 IEEE Global Engineering Education Conference (EDUCON) (pp. 563–570). IEEE.
[S039] Madan, A., Cebrian, M., Moturu, S., and Farrahi, K. (2011). Sensing the" health state" of a community. IEEE Pervasive Computing, 11(4), 36–45.
[S040] Sarkar, S., and Parnin, C. (2017, May). Characterizing and predicting mental fatigue during programming tasks. In 2017 IEEE/ACM 2nd International Workshop on Emotion Awareness in Software Engineering (SEmotion) (pp. 32–37). IEEE.
[S041] Zheng, Z., Wang, L., Cao, Y., Zhuang, Y., and Tao, X. (2019, December). Towards Non-Invasive Recognition of Developers’ Flow States with Computer Interaction Traces. In 2019 26th Asia-Pacific Software Engineering Conference (APSEC) (pp. 300–307). IEEE.
[S042] Palviainen, J., and Rezaei, P. P. (2015, September). The next level of user experience of cloud storage services: Supporting collaboration with social features. In 2015 24th Australasian Software Engineering Conference (pp. 175–184). IEEE.
[S043] Zangiacomi, A., Redaelli, C., Valentini, F., and Bernardelli, G. (2014, November). Design of interaction in a Virtual Environment for post-stroke rehabilitation: a cognitive perspective. In 2014 5th IEEE Conference on Cognitive Infocommunications (CogInfoCom) (pp. 167–172). IEEE.
[S044] Lederer, N., Grechenig, T., and Baranyi, R. (2014, May). unCUT: bridging the gap from paper diary cards towards mobile electronic monitoring solutions in borderline and self-injury. In 2014 IEEE 3nd International Conference on Serious Games and Applications for Health (SeGAH) (pp. 1–8). IEEE.
[S045] Kanjo, E., Kuss, D. J., and Ang, C. S. (2017). NotiMind: utilizing responses to smart phone notifications as affective sensors. IEEE Access, 5, 22023–22035.
[S046] Cornacchia, M., Ozcan, K., Zheng, Y., and Velipasalar, S. (2016). A survey on activity detection and classification using wearable sensors. IEEE Sensors Journal, 17(2), 386–403.
[S047] Nunes, D. S., Zhang, P., and Silva, J. S. (2015). A survey on human-in-the-loop applications towards an internet of all. IEEE Communications Surveys and Tutorials, 17(2), 944–965.
[S048] Schröder, S., Hirschl, J., and Reichl, P. (2018, May). Exploring the Interplay of Context and Interaction in the Field. In 2018 Tenth International Conference on Quality of Multimedia Experience (QoMEX) (pp. 1–6). IEEE.
[S049] Sinha, A., Gavas, R., Chatterjee, D., Das, R., and Sinharay, A. (2015, October). Dynamic assessment of learners’ mental state for an improved learning experience. In 2015 IEEE frontiers in education conference (FIE) (pp. 1–9). IEEE.
[S050] Fritz, T., and Müller, S. C. (2016, March). Leveraging biometric data to boost software developer productivity. In 2016 IEEE 23rd international conference on software analysis, evolution, and reengineering (SANER) (Vol. 5, pp. 66–77). IEEE.
[S051] Kimani, E., Rowan, K., McDuff, D., Czerwinski, M., and Mark, G. (2019, September). A conversational agent in support of productivity and wellbeing at work. In 2019 8th International Conference on Affective Computing and Intelligent Interaction (ACII) (pp. 1–7). IEEE.
[S052] Chen, Y., Mark, G., Ali, S., and Ma, X. (2017, August). Unpacking happiness: Lessons from smartphone photography among college students. In 2017 IEEE International Conference on Healthcare Informatics (ICHI) (pp. 429–438). IEEE.
[S053] Ko, A. J. (2017, May). A three-year participant observation of software startup software evolution. In 2017 IEEE/ACM 39th International Conference on Software Engineering: Software Engineering in Practice Track (ICSE-SEIP) (pp. 3–12). IEEE.
[S054] Ghandeharioun, A., Fedor, S., Sangermano, L., Ionescu, D., Alpert, J., Dale, C., … and Picard, R. (2017, October). Objective assessment of depressive symptoms with machine learning and wearable sensors data. In 2017 seventh international conference on affective computing and intelligent interaction (ACII) (pp. 325–332). IEEE.
[S055] Knappmeyer, M., Kiani, S. L., Reetz, E. S., Baker, N., and Tonjes, R. (2013). Survey of context provisioning middleware. IEEE Communications Surveys and Tutorials, 15(3), 1492–1519.
[S056] Moturu, S. T., Khayal, I., Aharony, N., Pan, W., and Pentland, A. (2011, January). Sleep, mood and sociability in a healthy population. In 2011 Annual International Conference of the IEEE Engineering in Medicine and Biology Society (pp. 5267–5270). IEEE.
[S057] Matthies, C., Huegle, J., Dürschmid, T., and Teusner, R. (2019, May). Attitudes, beliefs, and development data concerning agile software development practices. In 2019 IEEE/ACM 41st International Conference on Software Engineering: Software Engineering Education and Training (ICSE-SEET) (pp. 158–169). IEEE.
[S058] Klaas, V. C., Tröster, G., Büel, N., Walt, H., and Jenewein, J. (2017, October). Smart-phone based monitoring of cancer related fatigue. In 2017 IEEE 13th International Conference on Wireless and Mobile Computing, Networking and Communications (WiMob) (pp. 249–256). IEEE.
[S059] Ayzenberg, Y., and Picard, R. W. (2013). FEEL: A system for frequent event and electrodermal activity labeling. IEEE journal of biomedical and health informatics, 18(1), 266–277.
[S060] Rauschenberg, C., van Os, J., Goedhart, M., Schieveld, J. N., and Reininghaus, U. (2021). Bullying victimization and stress sensitivity in help-seeking youth: findings from an experience sampling study. European child & adolescent psychiatry, 30(4), 591.
[S061] Uink, B., Modecki, K. L., Barber, B. L., and Correia, H. M. (2018). Socioeconomically disadvantaged adolescents with elevated externalizing symptoms show heightened emotion reactivity to daily stress: An experience sampling study. Child Psychiatry and Human Development, 49(5), 741–756.
[S062] Atz, U. (2013). Evaluating experience sampling of stress in a single-subject research design. Personal and ubiquitous computing, 17(4), 639–652.
[S063] Lehtamo, S., Juuti, K., Inkinen, J., and Lavonen, J. (2018). Connection between academic emotions in situ and retention in the physics track: applying experience sampling method. International journal of STEM education, 5(1), 1–6.
[S064] Eddington, K. M., Burgin, C. J., Silvia, P. J., Fallah, N., Majestic, C., and Kwapil, T. R. (2017). The effects of psychotherapy for major depressive disorder on daily mood and functioning: a longitudinal experience sampling study. Cognitive therapy and research, 41(2), 266–277.
[S065] Sperry, S. H., Barrantes-Vidal, N., and Kwapil, T. R. (2018). The association of affective temperaments and bipolar spectrum psychopathology: An experience sampling study. Motivation and Emotion, 42(1), 126–136.
[S066] Gotink, R. A., Hermans, K. S., Geschwind, N., De Nooij, R., De Groot, W. T., and Speckens, A. E. (2016). Mindfulness and mood stimulate each other in an upward spiral: a mindful walking intervention using experience sampling. Mindfulness, 7(5), 1114–1122.
[S067] Fuller-Tyszkiewicz, M., Karvounis, T., Pemberton, R., Hartley-Clark, L., and Richardson, B. (2017). Determinants of depressive mood states in everyday life: An experience sampling study. Motivation and Emotion, 41(4), 510–521.
[S068] Chen, Y. W., Bundy, A., Cordier, R., Chien, Y. L., and Einfeld, S. (2016). The experience of social participation in everyday contexts among individuals with autism spectrum disorders: An experience sampling study. Journal of Autism and Developmental Disorders, 46(4), 1403–1414.
[S069] Bringsén, Å., Ejlertsson, G., and Andersson, I. H. (2011). Flow situations during everyday practice in a medical hospital ward. Results from a study based on experience sampling method. BMC nursing, 10(1), 1–9.
[S070] Beaty, R. E., Seli, P., and Schacter, D. L. (2019). Thinking about the past and future in daily life: an experience sampling study of individual differences in mental time travel. Psychological research, 83(4), 805–816.
[S071] Daros, A. R., Daniel, K. E., Meyer, M. J., Chow, P. I., Barnes, L. E., and Teachman, B. A. (2019). Impact of social anxiety and social context on college students’ emotion regulation strategy use: An experience sampling study. Motivation and Emotion, 43(5), 844–855.
[S072] O’Toole, M. S., Jensen, M. B., Fentz, H. N., Zachariae, R., and Hougaard, E. (2014). Emotion differentiation and emotion regulation in high and low socially anxious individuals: An experience-sampling study. Cognitive Therapy and Research, 38(4), 428–438.
[S073] Van Knippenberg, R. J., de Vugt, M. E., Ponds, R. W., Myin-Germeys, I., and Verhey, F. R. (2016). Dealing with daily challenges in dementia (deal-id study): effectiveness of the experience sampling method intervention’Partner in Sight’for spousal caregivers of people with dementia: design of a randomized controlled trial. BMC psychiatry, 16(1), 1–14.
[S074] Lawson, K. M. (2020). An Examination of Daily Experiences of Sexism and Reactivity among Women in US Male-Dominated Academic Majors Using Experience Sampling Methodology. Sex Roles, 83.
[S075] Hennig, T., and Lincoln, T. M. (2018). Sleeping paranoia away? An actigraphy and experience-sampling study with adolescents. Child Psychiatry and Human Development, 49(1), 63–72.
[S076] Grund, A., Grunschel, C., Bruhn, D., and Fries, S. (2015). Torn between want and should: An experience-sampling study on motivational conflict, well-being, self-control, and mindfulness. Motivation and Emotion, 39(4), 506–520.
[S077] Dawood, S., Hallquist, M. N., Pincus, A. L., Ram, N., Newman, M. G., Wilson, S. J., and Levy, K. N. (2020). Comparing Signal-Contingent and Event-Contingent Experience Sampling Ratings of Affect in a Sample of Psychotherapy Outpatients. Journal of psychopathology and behavioral assessment, 42(1), 13–24.
[S078] Goetz, T., Frenzel, A. C., Hall, N. C., Nett, U. E., Pekrun, R., and Lipnevich, A. A. (2014). Types of boredom: An experience sampling approach. Motivation and Emotion, 38(3), 401–419.
[S079] Bastiaansen, J. A., Meurs, M., Stelwagen, R., Wunderink, L., Schoevers, R. A., Wichers, M., and Oldehinkel, A. J. (2018). Self-monitoring and personalized feedback based on the experiencing sampling method as a tool to boost depression treatment: a protocol of a pragmatic randomized controlled trial (ZELF-i). BMC psychiatry, 18(1), 1–11.
[S080] Garrison, K. A., Pal, P., Rojiani, R., Dallery, J., O’Malley, S. S., and Brewer, J. A. (2015). A randomized controlled trial of smartphone-based mindfulness training for smoking cessation: a study protocol. BMC psychiatry, 15(1), 1–7.
[S081] Chin, B., Slutsky, J., Raye, J., and Creswell, J. D. Mindfulness Training Reduces Stress At Work: A Randomized Controlled Trial. Mindfulness (NY). 2019; 10 (4): 627–38.
[S082] Wray, T. B., Luo, X., Ke, J., Pérez, A. E., Carr, D. J., and Monti, P. M. (2019). Using smartphone survey data and machine learning to identify situational and contextual risk factors for HIV risk behavior among men who have sex with men who are not on PrEP. Prevention Science, 20(6), 904–913.
[S083] Senker, K., Fries, S., and Grund, A. (2020). Mindfulness in everyday life: between-and within-person relationships to motivational conflicts. Current Psychology, 1–16.
[S084] Gaggioli, A., Pioggia, G., Tartarisco, G., Baldus, G., Corda, D., Cipresso, P., and Riva, G. (2013). A mobile data collection platform for mental health research. Personal and Ubiquitous Computing, 17(2), 241–251.
[S085] Wohlfahrt-Laymann, J., Hermens, H., Villalonga, C., Vollenbroek-Hutten, M., and Banos, O. (2019). MobileCogniTracker. Journal of ambient intelligence and humanized computing, 10(6), 2143–2160.
[S086] Beymer, P. N., Rosenberg, J. M., Schmidt, J. A., and Naftzger, N. J. (2018). Examining relationships among choice, affect, and engagement in summer STEM programs. Journal of youth and adolescence, 47(6), 1178–1191.
[S087] Nylocks, K. M., Rafaeli, E., Bar-Kalifa, E., Flynn, J. J., and Coifman, K. G. (2019). Testing the influence of negative and positive emotion on future health-promoting behaviors in a community sample. Motivation and Emotion, 43(2), 285–298.
[S088] Bhayee, S., Tomaszewski, P., Lee, D. H., Moffat, G., Pino, L., Moreno, S., and Farb, N. A. (2016). Attentional and affective consequences of technology supported mindfulness training: a randomised, active control, efficacy trial. BMC psychology, 4(1), 1–14.
[S089] Milyavskaya, M., Saffran, M., Hope, N., and Koestner, R. (2018). Fear of missing out: prevalence, dynamics, and consequences of experiencing FOMO. Motivation and Emotion, 42(5), 725–737.
[S090] Glaros, A. G., Hanson, A. H., and Ryen, C. C. (2014). Headache and oral parafunctional behaviors. Applied psychophysiology and biofeedback, 39(1), 59–66.
[S091] Johnson, E. I., and Swendsen, J. D. (2015). Perceived social status and early adolescents’ responses to negative daily events. Journal of Child and Family Studies, 24(6), 1593–1604.
[S092] Linz, R., Pauly, R., Smallwood, J., and Engert, V. (2019). Mind-wandering content differentially translates from lab to daily life and relates to subjective stress experience. Psychological research, 1–11.
[S093] Warden, E. A., Plimpton, B., and Kvavilashvili, L. (2019). Absence of age effects on spontaneous past and future thinking in daily life. Psychological Research, 83(4), 727–746.
[S094] Debrot, A., Siegler, S., Klumb, P. L., and Schoebi, D. (2018). Daily work stress and relationship satisfaction: Detachment affects romantic couples’ interactions quality. Journal of Happiness Studies, 19(8), 2283–2301.
[S095] Armey, M. F., Nugent, N. R., and Crowther, J. H. (2012). An exploratory analysis of situational affect, early life stress, and nonsuicidal self-injury in college students. Journal of Child and Adolescent Trauma, 5(4), 327–343.
[S096] Krönke, K. M., Wolff, M., Mohr, H., Kräplin, A., Smolka, M. N., Bühringer, G., and Goschke, T. (2018). Monitor yourself! Deficient error-related brain activity predicts real-life self-control failures. Cognitive, Affective, and Behavioral Neuroscience, 18(4), 622–637.
[S097] Grund, A., and Carstens, C. A. (2019). Self-control motivationally reconsidered:“Acting” self-controlled is different to “being good” at self-control. Motivation and Emotion, 43(1), 63–81.
[S098] Chaieb, L., Derner, M., Leszczyński, M., and Fell, J. (2020). Modulation of Mind Wandering Using Auditory Beat Stimulation: a Pilot Study. Journal of Cognitive Enhancement, 4(1), 40–48.
[S099] Hofmann, W., Kotabe, H., and Luhmann, M. (2013). The spoiled pleasure of giving in to temptation. Motivation and Emotion, 37(4), 733–742.
[S100] Hilmert, C. J., Ode, S., Zielke, D. J., and Robinson, M. D. (2010). Blood pressure reactivity predicts somatic reactivity to stress in daily life. Journal of behavioral medicine, 33(4), 282–292.
[S101] Frissen, A., Lieverse, R., Drukker, M., Delespaul, P., Lataster, T., Myin-Germeys, I., and van Os, J. (2014). Evidence that childhood urban environment is associated with blunted stress reactivity across groups of patients with psychosis, relatives of patients and controls. Social psychiatry and psychiatric epidemiology, 49(10), 1579–1587.
[S102] Suelmann, H., Brouwers, A., and Snippe, E. (2018). Explaining variations in mindfulness levels in daily life. Mindfulness, 9(6), 1895–1906.
[S103] Litmanen, T., Lonka, K., Inkinen, M., Lipponen, L., and Hakkarainen, K. (2012). Capturing teacher students’ emotional experiences in context: does inquiry-based learning make a difference?. Instructional Science, 40(6), 1083–1101.
[S104] Berrios, R., Totterdell, P., and Kellett, S. (2018). Silver linings in the face of temptations: how mixed emotions promote self-control efforts in response to goal conflict. Motivation and Emotion, 42(6), 909–919.
[S105] Freire, T., Gomes, J., and Fonte, C. (2017). Adolescents’ Positive and Negative Affect and Relations With Alcohol Use: A Weekly Study. Child Indicators Research, 10(2), 525–543.
[S106] Hendriks, M., Ludwigs, K., and Veenhoven, R. (2016). Why are locals happier than internal migrants? The role of daily life. Social Indicators Research, 125(2), 481–508.
[S107] Jazaieri, H., McGonigal, K., Lee, I. A., Jinpa, T., Doty, J. R., Gross, J. J., and Goldin, P. R. (2018). Altering the trajectory of affect and affect regulation: The impact of compassion training. Mindfulness, 9(1), 283–293.
[S108] Menne-Lothmann, C., Jacobs, N., Derom, C., Thiery, E., van Os, J., and Wichers, M. (2012). Genetic and environmental causes of individual differences in daily life positive affect and reward experience and its overlap with stress-sensitivity. Behavior genetics, 42(5), 778–786.
[S109] South, S. C., and Miller, M. L. (2014). Measuring momentary stress, affect, and cognition: Relationships with the internalizing and externalizing spectra. Journal of Psychopathology and Behavioral Assessment, 36(1), 93–104.
[S110] Fogarty, F. A., Lu, L. M., Sollers, J. J., Krivoschekov, S. G., Booth, R. J., and Consedine, N. S. (2015). Why it pays to be mindful: trait mindfulness predicts physiological recovery from emotional stress and greater differentiation among negative emotions. Mindfulness, 6(2), 175–185.
[S111] Bachmann, O., Grunschel, C., and Fries, S. (2019). Multitasking and feeling good? Autonomy of additional activities predicts affect. Journal of Happiness Studies, 20(3), 899–918.
[S112] Saulin, A., Baumgartner, T., Gianotti, L. R., Hofmann, W., and Knoch, D. (2019). Frequency of helping friends and helping strangers is explained by different neural signatures. Cognitive, Affective, and Behavioral Neuroscience, 19(1), 177–186.
[S113] Pavani, J. B., Le Vigouroux, S., Kop, J. L., Congard, A., and Dauvier, B. (2016). Affect and affect regulation strategies reciprocally influence each other in daily life: The case of positive reappraisal, problem-focused coping, appreciation and rumination. Journal of Happiness Studies, 17(5), 2077–2095.
[S114] Chow, S. M., and Zhang, G. (2013). Nonlinear regime-switching state-space (RSSS) models. Psychometrika, 78(4), 740–768.
[S115] Blanke, E. S., and Brose, A. (2017). Mindfulness in daily life: A multidimensional approach. Mindfulness, 8(3), 737–750.
[S116] Kärner, T., and Kögler, K. (2016). Emotional states during learning situations and students’ self-regulation: process-oriented analysis of person-situation interactions in the vocational classroom. Empirical Research in Vocational Education and Training, 8(1), 1–23.
[S117] van Zundert, R. M., van Roekel, E., Engels, R. C., and Scholte, R. H. (2015). Reciprocal associations between adolescents’ night-time sleep and daytime affect and the role of gender and depressive symptoms. Journal of Youth and Adolescence, 44(2), 556–569.
[S118] Houben, M., Claes, L., Sleuwaegen, E., Berens, A., and Vansteelandt, K. (2018). Emotional reactivity to appraisals in patients with a borderline personality disorder: a daily life study. Borderline personality disorder and emotion dysregulation, 5(1), 1–13.
[S119] Bassi, M., and Delle Fave, A. (2012). Optimal experience and self-determination at school: Joining perspectives. Motivation and emotion, 36(4), 425–438.
[S120] Qin, X., Dust, S. B., DiRenzo, M. S., and Wang, S. (2019). Negative creativity in leader-follower relations: A daily investigation of leaders’ creative mindset, moral disengagement, and abusive supervision. Journal of Business and Psychology, 1–18.
[S121] Howell, R. T., Chenot, D., Hill, G., and Howell, C. J. (2011). Momentary happiness: The role of psychological need satisfaction. Journal of Happiness Studies, 12(1), 1–15.
[S122] Carlson, E. B., Field, N. P., Ruzek, J. I., Bryant, R. A., Dalenberg, C. J., Keane, T. M., and Spain, D. A. (2016). Advantages and psychometric validation of proximal intensive assessments of patient-reported outcomes collected in daily life. Quality of Life Research, 25(3), 507–516.
[S123] Harter, J. K., and Stone, A. A. (2012). Engaging and disengaging work conditions, momentary experiences and cortisol response. Motivation and Emotion, 36(2), 104–113.
[S124] Pot-Kolder, R., Veling, W., Geraets, C., and van der Gaag, M. (2016). Effect of virtual reality exposure therapy on social participation in people with a psychotic disorder (VRETp): study protocol for a randomized controlled trial. Trials, 17(1), 1–9.
[S125] Augustine, A. A., Hemenover, S. H., Larsen, R. J., and Shulman, T. E. (2010). Composition and consistency of the desired affective state: The role of personality and motivation. Motivation and Emotion, 34(2), 133–143.
[S126] Adams, P., Rabbi, M., Rahman, T., Matthews, M., Voida, A., Gay, G., … and Voida, S. (2014, May). Towards personal stress informatics: comparing minimally invasive techniques for measuring daily stress in the wild. In Proceedings of the 8th International Conference on Pervasive Computing Technologies for Healthcare (pp. 72–79).
[S127] Balters, S., Bernstein, M., and Paredes, P. E. (2019, May). On-road stress analysis for in-car interventions during the commute. In Extended Abstracts of the 2019 CHI Conference on Human Factors in Computing Systems (pp. 1–6).
[S128] Dietz, M., Aslan, I., Schiller, D., Flutura, S., Steinert, A., Klebbe, R., and André, E. (2019, May). Stress annotations from older adults-exploring the foundations for mobile ML-based health assistance. In Proceedings of the 13th EAI International Conference on Pervasive Computing Technologies for Healthcare (pp. 149–158).
[S129] Mathur, A., and Kawsar, F. (2017, September). Towards cognitive awareness: a mobile context modeling-and notification-based approach. In Proceedings of the 2017 ACM International Joint Conference on Pervasive and Ubiquitous Computing and Proceedings of the 2017 ACM International Symposium on Wearable Computers (pp. 977–981).
[S130] Paredes, P., Gilad-Bachrach, R., Czerwinski, M., Roseway, A., Rowan, K., and Hernandez, J. (2014, May). PopTherapy: Coping with stress through pop-culture. In Proceedings of the 8th International Conference on Pervasive Computing Technologies for Healthcare (pp. 109–117).
[S131] Kuutila, M., Mäntylä, M. V., Claes, M., Elovainio, M., and Adams, B. (2018, October). Using experience sampling to link software repositories with emotions and work well-being. In Proceedings of the 12th ACM/IEEE International Symposium on Empirical Software Engineering and Measurement (pp. 1–10). 1–15.
[S132] Kushlev, K., Cardoso, B., and Pielot, M. (2017, September). Too tense for candy crush: affect influences user engagement with proactively suggested content. In Proceedings of the 19th International Conference on Human-Computer Interaction with Mobile Devices and Services (pp. 1–6).
[S133] Ciman, M., Wac, K., and Gaggi, O. (2015, May). iSenseStress: Assessing stress through human-smartphone interaction analysis. In 2015 9th International conference on pervasive computing technologies for healthcare (PervasiveHealth) (pp. 84–91). IEEE.
[S134] King, Z., Moskowitz, J., Wakschlag, L., and Alshurafa, N. (2018, October). Predicting Perceived Stress Through Mirco-EMAs and a Flexible Wearable ECG Device. In Proceedings of the 2018 ACM International Joint Conference and 2018 International Symposium on Pervasive and Ubiquitous Computing and Wearable Computers (pp. 106–109).
[S135] Zhang, Z., Zheng, J., Li, Z., and Zhang, L. (2020, January). Job Insecurity and Daily Emotional Exhaustion: An Experience Sampling Method Approach. In Proceedings of the 2020 4th International Conference on Management Engineering, Software Engineering and Service Sciences (pp. 271–276).
[S136] Meschtscherjakov, A., Wilfinger, D., Osswald, S., Perterer, N., and Tscheligi, M. (2012, October). Trip experience sampling: Assessing driver experience in the field. In Proceedings of the 4th international conference on automotive user interfaces and interactive vehicular applications (pp. 225–232).
[S137] Mark, G., Iqbal, S. T., Czerwinski, M., and Johns, P. (2014, April). Bored mondays and focused afternoons: the rhythm of attention and online activity in the workplace. In Proceedings of the SIGCHI Conference on Human Factors in Computing Systems (pp. 3025–3034).
[S138] Tollmar, K., and Huang, C. (2015, August). Boosting mobile experience sampling with social media. In Proceedings of the 17th International Conference on Human-Computer Interaction with Mobile Devices and Services (pp. 525–530).
[S139] Guillou, H., Chow, K., Fritz, T., and McGrenere, J. (2020, April). Is Your Time Well Spent? Reflecting on Knowledge Work More Holistically. In Proceedings of the 2020 CHI Conference on Human Factors in Computing Systems (pp. 1–9).
[S140] Epp, C., Lippold, M., and Mandryk, R. L. (2011, May). Identifying emotional states using keystroke dynamics. In Proceedings of the sigchi conference on human factors in computing systems (pp. 715–724).
[S141] Spathis, D., Servia-Rodriguez, S., Farrahi, K., Mascolo, C., and Rentfrow, J. (2019, May). Passive mobile sensing and psychological traits for large scale mood prediction. In Proceedings of the 13th EAI International Conference on Pervasive Computing Technologies for Healthcare (pp. 272–281).
[S142] Panger, G. (2018). People tend to wind down, not up, when they browse social media. Proceedings of the ACM on Human-Computer Interaction, 2(CSCW), 1–29.
[S143] Mark, G., Iqbal, S., Czerwinski, M., and Johns, P. (2014, February). Capturing the mood: facebook and face-to-face encounters in the workplace. In Proceedings of the 17th ACM conference on Computer supported cooperative work and social computing (pp. 1082–1094).
[S144] Hassib, M., Khamis, M., Friedl, S., Schneegass, S., and Alt, F. (2017, November). Brainatwork: Logging cognitive engagement and tasks in the workplace using electroencephalography. In Proceedings of the 16th international conference on mobile and ubiquitous multimedia (pp. 305–310).
[S145] Mark, G., Iqbal, S., Czerwinski, M., and Johns, P. (2015, February). Focused, aroused, but so distractible: Temporal perspectives on multitasking and communications. In Proceedings of the 18th ACM Conference on Computer Supported Cooperative Work and Social Computing (pp. 903–916).
[S146] Ghandeharioun, A., and Picard, R. (2017, May). BrightBeat: effortlessly influencing breathing for cultivating calmness and focus. In Proceedings of the 2017 CHI conference extended abstracts on human factors in computing systems (pp. 1624–1631).
[S147] Kushlev, K., Cardoso, B., and Pielot, M. (2017, September). Too tense for candy crush: affect influences user engagement with proactively suggested content. In Proceedings of the 19th International Conference on Human-Computer Interaction with Mobile Devices and Services (pp. 1–6).
[S148] Ghosh, S., Ganguly, N., Mitra, B., and De, P. (2017, September). Tapsense: Combining self-report patterns and typing characteristics for smartphone based emotion detection. In Proceedings of the 19th International Conference on Human-Computer Interaction with Mobile Devices and Services (pp. 1–12).
[S149] Kuzminykh, A., and Lank, E. (2019). How much is too much? understanding the information needs of parents of young children. Proceedings of the ACM on Interactive, Mobile, Wearable and Ubiquitous Technologies, 3(2), 1–21.
[S150] Niforatos, E., Karapanos, E., Langheinrich, M., Wurhofer, D., Krischkowsky, A., Obrist, M., and Tscheligi, M. (2015, September). eMotion: retrospective in-car user experience evaluation. In Adjunct Proceedings of the 7th International Conference on Automotive User Interfaces and Interactive Vehicular Applications (pp. 118–123).
[S151] Hernandez, N., Demiray, B., Arnrich, B., and Favela, J. (2019, May). An Exploratory Study to Detect Temporal Orientation Using Bluetooth’s sensor. In Proceedings of the 13th EAI International Conference on Pervasive Computing Technologies for Healthcare (pp. 292–297).
[S152] Ghosh, S., Chauhan, V., Ganguly, N., Mitra, B., and De, P. (2015, September). Impact of experience sampling methods on tap pattern based emotion recognition. In Adjunct Proceedings of the 2015 ACM International Joint Conference on Pervasive and Ubiquitous Computing and Proceedings of the 2015 ACM International Symposium on Wearable Computers (pp. 713–722).
[S153] J. Rough, D., Quigley, A. (2015, August). End-User Development of Experience Sampling Smartphone Apps -Recommendations and Requirements. Proceedings of the ACM on Interactive, Mobile, Wearable and Ubiquitous Technologies (pp. 1–19).
[S154] Barathi, S. C., Proulx, M., O’Neill, E., and Lutteroth, C. (2020, April). Affect recognition using psychophysiological correlates in high intensity VR exergaming. In Proceedings of the 2020 CHI Conference on Human Factors in Computing Systems (pp. 1–15).
[S155] Rector, K., and Hailpern, J. (2014, April). MinEMail: SMS alert system for managing critical emails. In Proceedings of the SIGCHI conference on Human factors in computing systems (pp. 783–792).
[S156] Vescovi, M., Perentis, C., Leonardi, C., Lepri, B., and Moiso, C. (2014, September). My data store: toward user awareness and control on personal data. In Proceedings of the 2014 ACM International Joint Conference on Pervasive and Ubiquitous Computing: Adjunct Publication (pp. 179–182).
[S157] Buschek, D., Hartmann, F., Von Zezschwitz, E., De Luca, A., and Alt, F. (2016, May). Snapapp: Reducing authentication overhead with a time-constrained fast unlock option. In Proceedings of the 2016 CHI Conference on Human Factors in Computing Systems (pp. 3736–3747).
[S158] Pfleging, B., Schneegass, S., Meschtscherjakov, A., and Tscheligi, M. (2014, September). Experience Maps: Experience-Enhanced Routes for Car Navigation. In Adjunct Proceedings of the 6th International Conference on Automotive User Interfaces and Interactive Vehicular Applications (pp. 1–6).
[S159] Musthag, M., Raij, A., Ganesan, D., Kumar, S., and Shiffman, S. (2011, September). Exploring micro-incentive strategies for participant compensation in high-burden studies. In Proceedings of the 13th international conference on Ubiquitous computing (pp. 435–444).
[S160] Karlesky, M., and Isbister, K. (2014, February). Designing for the physical margins of digital workspaces: fidget widgets in support of productivity and creativity. In Proceedings of the 8th international conference on tangible, embedded and embodied interaction (pp. 13–20).
[S161] Wang, Y., and Mark, G. (2018, April). The context of college students’ facebook use and academic performance: An empirical study. In Proceedings of the 2018 chi conference on human factors in computing systems (pp. 1–11).
[S162] Servia-Rodríguez, S., Rachuri, K. K., Mascolo, C., Rentfrow, P. J., Lathia, N., and Sandstrom, G. M. (2017, April). Mobile sensing at the service of mental well-being: a large-scale longitudinal study. In Proceedings of the 26th International Conference on World Wide Web (pp. 103–112).
[S163] Meyer, A. N., Murphy, G. C., Zimmermann, T., and Fritz, T. (2017). Design recommendations for self-monitoring in the workplace: Studies in software development. Proceedings of the ACM on Human-Computer Interaction, 1(CSCW), 1–24.
[S164] van Berkel, N., Goncalves, J., Koval, P., Hosio, S., Dingler, T., Ferreira, D., and Kostakos, V. (2019, May). Context-informed scheduling and analysis: improving accuracy of mobile self-reports. In Proceedings of the 2019 CHI Conference on Human Factors in Computing Systems (pp. 1–12).
[S165] Patel, S., Mahamuni, R., Singh, M., Clarance, D., Duggirala, M., Sharma, S., … and Balaraman, V. (2016, March). Mining Multi-source Data to Study Workplace Activity Patterns. In Proceedings of the 3rd IKDD Conference on Data Science, 2016 (pp. 1–2).
[S166] Paruthi, G., Raj, S., Gupta, A., Huang, C. C., Chang, Y. J., and Newman, M. W. (2017, September). HEED: situated and distributed interactive devices for self-reporting. In Proceedings of the 2017 ACM International Joint Conference on Pervasive and Ubiquitous Computing and Proceedings of the 2017 ACM International Symposium on Wearable Computers (pp. 181–184).
[S167] Li, C. T., Cao, J., and Li, T. M. (2016, September). Eustress or distress: An empirical study of perceived stress in everyday college life. In Proceedings of the 2016 ACM International Joint Conference on Pervasive and Ubiquitous Computing: Adjunct (pp. 1209–1217).
[S168] Khamis, M., Baier, A., Henze, N., Alt, F., and Bulling, A. (2018, April). Understanding face and eye visibility in front-facing cameras of smartphones used in the wild. In Proceedings of the 2018 CHI Conference on Human Factors in Computing Systems (pp. 1–12).
[S169] Mehrotra, A., and Musolesi, M. (2017, June). Designing effective movement digital biomarkers for unobtrusive emotional state mobile monitoring. In Proceedings of the 1st Workshop on Digital Biomarkers (pp. 3–8).
[S170] Pielot, M., Cardoso, B., Katevas, K., Serrà, J., Matic, A., and Oliver, N. (2017). Beyond interruptibility: Predicting opportune moments to engage mobile phone users. Proceedings of the ACM on Interactive, Mobile, Wearable and Ubiquitous Technologies, 1(3), 1–25.
[S171] Darvariu, V. A., Convertino, L., Mehrotra, A., and Musolesi, M. (2020). Quantifying the relationships between everyday objects and emotional states through deep learning based image analysis using smartphones. Proceedings of the ACM on Interactive, Mobile, Wearable and Ubiquitous Technologies, 4(1), 1–21.
[S172] Pielot, M., Church, K., and De Oliveira, R. (2014, September). An in-situ study of mobile phone notifications. In Proceedings of the 16th international conference on Human-computer interaction with mobile devices and services (pp. 233–242).
[S173] Pielot, M., Dingler, T., Pedro, J. S., and Oliver, N. (2015, September). When attention is not scarce-detecting boredom from mobile phone usage. In Proceedings of the 2015 ACM international joint conference on pervasive and ubiquitous computing (pp. 825–836).
[S174] Rachuri, K. K., and Mascolo, C. (2011, September). Smart phone based systems for social psychological research: Challenges and design guidelines. In Proceedings of the 3rd ACM Workshop on Wireless of the Students, by the Students, for the Students (pp. 21–24).
[S175] Osmani, V., Maxhuni, A., Grünerbl, A., Lukowicz, P., Haring, C., and Mayora, O. (2013, December). Monitoring activity of patients with bipolar disorder using smart phones. In Proceedings of International Conference on Advances in Mobile Computing and Multimedia (pp. 85–92).
[S176] Hiniker, A., Froehlich, J. E., Zhang, M., and Beneteau, E. (2019, May). Anchored audio sampling: A seamless method for exploring children’s thoughts during deployment studies. In Proceedings of the 2019 CHI Conference on Human Factors in Computing Systems (pp. 1–13).
[S177] Mark, G., Czerwinski, M., Iqbal, S., and Johns, P. (2016, April). Workplace indicators of mood: Behavioral and cognitive correlates of mood among information workers. In Proceedings of the 6th International Conference on Digital Health Conference (pp. 29–36).
[S178] Xu, A., Biehl, J., Rieffel, E., Turner, T., and van Melle, W. (2012, May). Learning how to feel again: Towards affective workplace presence and communication technologies. In Proceedings of the SIGCHI Conference on Human Factors in Computing Systems (pp. 839–848).
[S179] Wurhofer, D., Krischkowsky, A., Obrist, M., Karapanos, E., Niforatos, E., and Tscheligi, M. (2015, September). Everyday commuting: prediction, actual experience and recall of anger and frustration in the car. In Proceedings of the 7th International Conference on Automotive User Interfaces and Interactive Vehicular Applications (pp. 233–240).
[S180] Karlesky, M., and Isbister, K. (2016, October). Understanding fidget widgets: Exploring the design space of embodied self-regulation. In Proceedings of the 9th Nordic Conference on Human-Computer Interaction (pp. 1–10).
[S181] Mehrotra, A., Pejovic, V., Vermeulen, J., Hendley, R., and Musolesi, M. (2016, May). My phone and me: understanding people’s receptivity to mobile notifications. In Proceedings of the 2016 CHI conference on human factors in computing systems (pp. 1021–1032).
[S182] Liu, F., Dabbish, L., and Kaufman, G. (2017). Supporting social interactions with an expressive heart rate sharing application. Proceedings of the ACM on Interactive, Mobile, Wearable and Ubiquitous Technologies, 1(3), 1–26.
[S183] Mehrotra, A., Tsapeli, F., Hendley, R., and Musolesi, M. (2017). MyTraces: Investigating correlation and causation between users’ emotional states and mobile phone interaction. Proceedings of the ACM on Interactive, Mobile, Wearable and Ubiquitous Technologies, 1(3), 1–21.
[S184] Ferreira, D., Goncalves, J., Kostakos, V., Barkhuus, L., and Dey, A. K. (2014, September). Contextual experience sampling of mobile application micro-usage. In Proceedings of the 16th international conference on Human-computer interaction with mobile devices and services (pp. 91–100).
[S185] Hahn, N., Iqbal, S. T., and Teevan, J. (2019, May). Casual microtasking: Embedding microtasks in Facebook. In Proceedings of the 2019 CHI Conference on Human Factors in Computing Systems (pp. 1–9).
[S186] Williams, A. C., Kaur, H., Mark, G., Thompson, A. L., Iqbal, S. T., and Teevan, J. (2018, April). Supporting workplace detachment and reattachment with conversational intelligence. In Proceedings of the 2018 CHI Conference on Human Factors in Computing Systems (pp. 1–13).
[S187] Ellison, W. D., Trahan, A. C., Pinzon, J. C., Gillespie, M. E., Simmons, L. M., and King, K. Y. (2020). For whom, and for what, is experience sampling more accurate than retrospective report?. Personality and Individual Differences, 163, 110071.
[S188] Gerritsen, C., Bagby, R. M., Sanches, M., Kiang, M., Maheandiran, M., Prce, I., and Mizrahi, R. (2019). Stress precedes negative symptom exacerbations in clinical high risk and early psychosis: A time-lagged experience sampling study. Schizophrenia research, 210, 52–58.
[S189] Chun, C., Gross, G., Mielock, A., and Kwapil, T. (2020). Aberrant salience predicts psychotic-like experiences in daily life: An experience sampling study. Schizophrenia research, 220, 218–224.
[S190] D’Arcy, J., and Teh, P. L. (2019). Predicting employee information security policy compliance on a daily basis: The interplay of security-related stress, emotions, and neutralization. Information and Management, 56(7), 103151.
[S191] Duvenage, M., Correia, H., Uink, B., Barber, B. L., Donovan, C. L., and Modecki, K. L. (2020). Technology can sting when reality bites: Adolescents’ frequent online coping is ineffective with momentary stress. Computers in Human Behavior, 102, 248–259.
[S192] Moeller, J., Brackett, M. A., Ivcevic, Z., and White, A. E. (2020). High school students’ feelings: Discoveries from a large national survey and an experience sampling study. Learning and Instruction, 66, 101301.
[S193] Greene, T. (2018). Do acute dissociation reactions predict subsequent posttraumatic stress and growth? A prospective experience sampling method study. Journal of anxiety disorders, 57, 1–6.
[S194] Weiss, M., Razinskas, S., Backmann, J., and Hoegl, M. (2018). Authentic leadership and leaders’ mental well-being: An experience sampling study. The Leadership Quarterly, 29(2), 309–321.
[S195] Bahlinger, K., Lincoln, T. M., Krkovic, K., and Clamor, A. (2020). Linking psychophysiological adaptation, emotion regulation, and subjective stress to the occurrence of paranoia in daily life. Journal of Psychiatric Research, 130, 152–159.
[S196] Schlotz, W. (2019). Investigating associations between momentary stress and cortisol in daily life: What have we learned so far?. Psychoneuroendocrinology, 105, 105–116.
[S197] Greene, T., Gelkopf, M., Grinapol, S., Werbeloff, N., Carlson, E., and Lapid, L. (2017). Trajectories of traumatic stress symptoms during conflict: A latent class growth analysis. Journal of affective disorders, 220, 24–30.
[S198] van Roekel, E., Ha, T., Verhagen, M., Kuntsche, E., Scholte, R. H., and Engels, R. C. (2015). Social stress in early adolescents’ daily lives: Associations with affect and loneliness. Journal of adolescence, 45, 274–283.
[S199] van Duin, E. D., Vaessen, T., Kasanova, Z., Viechtbauer, W., Reininghaus, U., Saalbrink, P., … and Myin-Germeys, I. (2019). Lower cortisol levels and attenuated cortisol reactivity to daily-life stressors in adults with 22q11. 2 deletion syndrome. Psychoneuroendocrinology, 106, 85–94.
[S200] Bos, F. M., Blaauw, F. J., Snippe, E., Van der Krieke, L., De Jonge, P., and Wichers, M. (2018). Exploring the emotional dynamics of subclinically depressed individuals with and without anhedonia: an experience sampling study. Journal of affective disorders, 228, 186–193.
[S201] Chong, S., Kim, Y. J., Lee, H. W., Johnson, R. E., and Lin, S. H. J. (2020). Mind your own break! The interactive effect of workday respite activities and mindfulness on employee outcomes via affective linkages. Organizational Behavior and Human Decision Processes, 159, 64–77.
[S202] MacIntyre, P. D., Ross, J., Talbot, K., Mercer, S., Gregersen, T., and Banga, C. A. (2019). Stressors, personality and wellbeing among language teachers. System, 82, 26–38.
[S203] Vaessen, T., Kasanova, Z., Hernaus, D., Lataster, J., Collip, D., van Nierop, M., and Myin-Germeys, I. (2018). Overall cortisol, diurnal slope, and stress reactivity in psychosis: An experience sampling approach. Psychoneuroendocrinology, 96, 61–68.
[S204] Zhang, C., Smolders, K. C., Lakens, D., and IJsselsteijn, W. A. (2018). Two experience sampling studies examining the variation of self-control capacity and its relationship with core affect in daily life. Journal of Research in Personality, 74, 102–113.
[S205] van Winkel, M., Nicolson, N. A., Wichers, M., Viechtbauer, W., Myin-Germeys, I., and Peeters, F. (2015). Daily life stress reactivity in remitted versus non-remitted depressed individuals. European Psychiatry, 30(4), 441–447.
[S206] Sperry, S. H., and Kwapil, T. R. (2017). What can daily life assessment tell us about the bipolar spectrum? Psychiatry research, 252, 51–56.
[S207] Pos, K., de Wit, I. E., van Dijk, F. A., Bartels-Velthuis, A. A., Bruggeman, R., Meijer, C. J., … and van Winkel, R. (2017). An experience sampling study on the ecological validity of the SWN-20: Indication that subjective well-being is associated with momentary affective states above and beyond psychosis susceptibility. Psychiatry research, 258, 234–238.
[S208] Havermans, R., Nicolson, N. A., Berkhof, J., and deVries, M. W. (2010). Mood reactivity to daily events in patients with remitted bipolar disorder. Psychiatry research, 179(1), 47–52.
[S209] Jones, D. R., Lehman, B. J., Kirsch, J. A., and Hennessy, K. G. (2017). Pessimism moderates negative emotional responses to naturally occurring stress. Journal of Research in Personality, 69, 180–190.
[S210] Offer, S. (2014). Time with children and employed parents’ emotional well-being. Social science research, 47, 192–203.
[S211] Prem, R., Kubicek, B., Diestel, S., and Korunka, C. (2016). Regulatory job stressors and their within-person relationships with ego depletion: The roles of state anxiety, self-control effort, and job autonomy. Journal of Vocational Behavior, 92, 22–32.
[S212] Vaessen, T., Viechtbauer, W., van der Steen, Y., Gayer-Anderson, C., Kempton, M. J., Valmaggia, L., … and Myin-Germeys, I. (2019). Recovery from daily-life stressors in early and chronic psychosis. Schizophrenia research, 213, 32–39.
[S213] Sitko, K., Varese, F., Sellwood, W., Hammond, A., and Bentall, R. (2016). The dynamics of attachment insecurity and paranoid thoughts: An experience sampling study. Psychiatry research, 246, 32–38.
[S214] Yang, C. H., and Conroy, D. E. (2018). Momentary negative affect is lower during mindful movement than while sitting: an experience sampling study. Psychology of sport and exercise, 37, 109–116.
[S215] van Winkel, M., Peeters, F., van Winkel, R., Kenis, G., Collip, D., Geschwind, N., … and Wichers, M. (2014). Impact of variation in the BDNF gene on social stress sensitivity and the buffering impact of positive emotions: replication and extension of a gene–environment interaction. European Neuropsychopharmacology, 24(6), 930–938.
[S216] Krkovic, K., Krink, S., and Lincoln, T. M. (2018). Emotion regulation as a moderator of the interplay between self-reported and physiological stress and paranoia. European Psychiatry, 49, 43–49.
[S217] van Nierop, M., Lecei, A., Myin-Germeys, I., Collip, D., Viechtbauer, W., Jacobs, N., … and van Winkel, R. (2018). Stress reactivity links childhood trauma exposure to an admixture of depressive, anxiety, and psychosis symptoms. Psychiatry research, 260, 451–457.
[S218] Engert, V., Kok, B. E., Puhlmann, L. M., Stalder, T., Kirschbaum, C., Apostolakou, F., … and Singer, T. (2018). Exploring the multidimensional complex systems structure of the stress response and its relation to health and sleep outcomes. Brain, behavior, and immunity, 73, 390–402.
[S219] Gevonden, M., Myin-Germeys, I., Wichers, M., Booij, J., van den Brink, W., van Winkel, R., and Selten, J. P. (2016). Reactivity to social stress in ethnic minority men. Psychiatry Research, 246, 629–636.
[S220] Kackar, H. Z., Shumow, L., Schmidt, J. A., and Grzetich, J. (2011). Age and gender differences in adolescents’ homework experiences. Journal of Applied Developmental Psychology, 32(2), 70–77.
[S221] Westermann, S., Grezellschak, S., Oravecz, Z., Moritz, S., Lüdtke, T., and Jansen, A. (2017). Untangling the complex relationships between symptoms of schizophrenia and emotion dynamics in daily life: Findings from an experience sampling pilot study. Psychiatry research, 257, 514–518.
[S222] Offer, S. (2013). Assessing the relationship between family mealtime communication and adolescent emotional well-being using the experience sampling method. Journal of Adolescence, 36(3), 577–585.
[S223] Booij, S. H., Snippe, E., Jeronimus, B. F., Wichers, M., and Wigman, J. T. (2018). Affective reactivity to daily life stress: Relationship to positive psychotic and depressive symptoms in a general population sample. Journal of affective disorders, 225, 474–481.
[S224] Johnson, J. A., Miller, M. L., Lynam, D. R., and South, S. C. (2012). Five-Factor Model facets differentially predict in-the-moment affect and cognitions. Journal of Research in Personality, 46(6), 752–759.
[S225] Skalina, L. M., Gunthert, K. C., Ahrens, A. H., and Wenze, S. J. (2015). Neuroticism and momentary differentiation of positive and negative affect. Personality and Individual Differences, 75, 165–169.
[S226] Havermans, R., Nicolson, N. A., Berkhof, J., and deVries, M. W. (2011). Patterns of salivary cortisol secretion and responses to daily events in patients with remitted bipolar disorder. Psychoneuroendocrinology, 36(2), 258–265.
[S227] Kroencke, L., Harari, G. M., Katana, M., and Gosling, S. D. (2019). Personality trait predictors and mental well-being correlates of exercise frequency across the academic semester. Social Science and Medicine, 236, 112400.
[S228] Corcoran, C. M., Smith, C., McLaughlin, D., Auther, A., Malaspina, D., and Cornblatt, B. (2012). HPA axis function and symptoms in adolescents at clinical high risk for schizophrenia. Schizophrenia research, 135(1–3), 170–174.
[S229] McKee, K., Russell, M., Mennis, J., Mason, M., and Neale, M. (2020). Emotion regulation dynamics predict substance use in high-risk adolescents. Addictive behaviors, 106, 106374.
[S230] Walsh, M. A., Brown, L. H., Barrantes-Vidal, N., and Kwapil, T. R. (2013). The expression of affective temperaments in daily life. Journal of Affective Disorders, 145(2), 179–186.
[S231] Tully, L. M., Lincoln, S. H., and Hooker, C. I. (2014). Lateral prefrontal cortex activity during cognitive control of emotion predicts response to social stress in schizophrenia. NeuroImage: Clinical, 6, 43–53.
[S232] Hoorelbeke, K., Van den Bergh, N., Wichers, M., and Koster, E. H. (2019). Between vulnerability and resilience: A network analysis of fluctuations in cognitive risk and protective factors following remission from depression. Behaviour research and therapy, 116, 1–9.
[S233] Gijzel, S. M., Rector, J., van Meulen, F. B., van Der Loeff, R. S., van de Leemput, I. A., Scheffer, M., … and Melis, R. J. (2020). Measurement of dynamical resilience indicators improves the prediction of recovery following hospitalization in older adults. Journal of the American Medical Directors Association, 21(4), 525–530.
[S234] Balasundaram, A. P., Athens, J., Schneiders, A. G., McCrory, P., and Sullivan, S. J. (2017). Psychological and lifestyle factors that influence the serial reporting of postconcussion-like symptoms in a non-concussed population. PMandR, 9(9), 866–873.
[S235] Huffziger, S., Ebner-Priemer, U., Zamoscik, V., Reinhard, I., Kirsch, P., and Kuehner, C. (2013). Effects of mood and rumination on cortisol levels in daily life: An ambulatory assessment study in remitted depressed patients and healthy controls. Psychoneuroendocrinology, 38(10), 2258–2267.
[S236] Reininghaus, U., Kempton, M. J., Valmaggia, L., Craig, T. K., Garety, P., Onyejiaka, A., … and Morgan, C. (2016). Stress sensitivity, aberrant salience, and threat anticipation in early psychosis: an experience sampling study. Schizophrenia bulletin, 42(3), 712–722.
[S237] Bono, J. E., Glomb, T. M., Shen, W., Kim, E., and Koch, A. J. (2013). Building positive resources: Effects of positive events and positive reflection on work stress and health. Academy of Management Journal, 56(6), 1601–1627.
[S238] Adams, P., Rabbi, M., Rahman, T., Matthews, M., Voida, A., Gay, G., … and Voida, S. (2014, May). Towards personal stress informatics: comparing minimally invasive techniques for measuring daily stress in the wild. In Proceedings of the 8th International Conference on Pervasive Computing Technologies for Healthcare (pp. 72–79).
[S239] Lardinois, M., Lataster, T., Mengelers, R., Van Os, J., and Myin-Germeys, I. (2011). Childhood trauma and increased stress sensitivity in psychosis. Acta Psychiatrica Scandinavica, 123(1), 28–35.
[S240] Greene, T., Gelkopf, M., Fried, E. I., Robinaugh, D. J., and Lapid Pickman, L. (2020). Dynamic network analysis of negative emotions and DSM-5 posttraumatic stress disorder symptom clusters during conflict. Journal of traumatic stress, 33(1), 72–83.
[S241] Duvenage, M., Correia, H., Uink, B., Barber, B. L., Donovan, C. L., and Modecki, K. L. (2020). Technology can sting when reality bites: Adolescents’ frequent online coping is ineffective with momentary stress. Computers in Human Behavior, 102, 248–259.
[S242] Collip, D., Nicolson, N. A., Lardinois, M., Lataster, T., Van Os, J., and Myin-Germeys, I. (2011). Daily cortisol, stress reactivity and psychotic experiences in individuals at above average genetic risk for psychosis. Psychological medicine, 41(11), 2305–2315.
[S243] Sang, B., Pan, T., Deng, X., and Zhao, X. (2018). Be cool with academic stress: the association between emotional states and regulatory strategies among Chinese adolescents. Educational Psychology, 38(1), 38–53.
[S244] Palmier-Claus, J. E., Dunn, G., and Lewis, S. W. (2012). Emotional and symptomatic reactivity to stress in individuals at ultra-high risk of developing psychosis. Psychological medicine, 42(5), 1003–1012.
[S245] Cristóbal-Narváez, P., Sheinbaum, T., Ballespí, S., Mitjavila, M., Myin-Germeys, I., Kwapil, T. R., and Barrantes-Vidal, N. (2016). Impact of adverse childhood experiences on psychotic-like symptoms and stress reactivity in daily life in nonclinical young adults. PloS one, 11(4), e0153557.
[S246] Kimhy, D., Delespaul, P., Ahn, H., Cai, S., Shikhman, M., Lieberman, J. A., … and Sloan, R. P. (2010). Concurrent measurement of “real-world” stress and arousal in individuals with psychosis: assessing the feasibility and validity of a novel methodology. Schizophrenia bulletin, 36(6), 1131–1139.
[S247] Collip, D., Wigman, J. T., Myin-Germeys, I., Jacobs, N., Derom, C., Thiery, E., … and van Os, J. (2013). From epidemiology to daily life: linking daily life stress reactivity to persistence of psychotic experiences in a longitudinal general population study. PloS one, 8(4), e62688.
[S248] Lataster, T., Collip, D., Lardinois, M., Van Os, J., and Myin-Germeys, I. (2010). Evidence for a familial correlation between increased reactivity to stress and positive psychotic symptoms. Acta Psychiatrica Scandinavica, 122(5), 395–404.
[S249] Linz, R., Singer, T., and Engert, V. (2018). Interactions of momentary thought content and subjective stress predict cortisol fluctuations in a daily life experience sampling study. Scientific reports, 8(1), 1–11.
[S250] Lataster, T., Valmaggia, L., Lardinois, M., Van Os, J., and Myin-Germeys, I. (2013). Increased stress reactivity: a mechanism specifically associated with the positive symptoms of psychotic disorder. Psychological medicine, 43(7), 1389–1400.
[S251] Klippel, A., Viechtbauer, W., Reininghaus, U., Wigman, J., van Borkulo, C., MERGE, … and Wichers, M. (2018). The cascade of stress: a network approach to explore differential dynamics in populations varying in risk for psychosis. Schizophrenia Bulletin, 44(2), 328–337.
[S252] van der Steen, Y., Gimpel-Drees, J., Lataster, T., Viechtbauer, W., Simons, C. J. P., Lardinois, M., … and Myin-Germeys, I. (2017). Clinical high risk for psychosis: the association between momentary stress, affective and psychotic symptoms. Acta Psychiatrica Scandinavica, 136(1), 63–73.
[S253] Collip, D., van Winkel, R., Peerbooms, O., Lataster, T., Thewissen, V., Lardinois, M., … and Myin-Germeys, I. (2011). COMT Val158Met–stress interaction in psychosis: role of background psychosis risk. CNS neuroscience and therapeutics, 17(6), 612–619.
[S254] van Winkel, M., Peeters, F., van Winkel, R., Kenis, G., Collip, D., Geschwind, N., … and Wichers, M. (2014). Impact of variation in the BDNF gene on social stress sensitivity and the buffering impact of positive emotions: replication and extension of a gene–environment interaction. European Neuropsychopharmacology, 24(6), 930–938.
[S255] Glaros, A. G., Marszalek, J. M., and Williams, K. B. (2016). Longitudinal multilevel modeling of facial pain, muscle tension, and stress. Journal of dental research, 95(4), 416–422.
[S256] Pacella, M. L., Girard, J. M., Wright, A. G., Suffoletto, B., and Callaway, C. W. (2018). The association between daily posttraumatic stress symptoms and pain over the first 14 days after injury: An experience sampling study. Academic emergency medicine, 25(8), 844–855.
[S257] Myin-Germeys, I. (2014). Psychotic reactivity to daily life stress and the dopamine system: a study combining experience sampling and [18F] fallypride positron emission tomography. Early Interventions in Psychiatry, 8, 37–37.
[S258] van Knippenberg, R. J., de Vugt, M. E., Ponds, R. W., Verhey, F. R., and Myin-Germeys, I. (2018). Emotional reactivity to daily life stress in spousal caregivers of people with dementia: An experience sampling study. PloS one, 13(4), e0194118.
[S259] Chan, Y., So, S. H. W., Mak, A. D. P., Siah, K. T. H., Chan, W., and Wu, J. C. (2019). The temporal relationship of daily life stress, emotions, and bowel symptoms in irritable bowel syndrome—Diarrhea subtype: A smartphone-based experience sampling study. Neurogastroenterology and Motility, 31(3), e13514.
[S260] Palmier-Claus, J. E., Dunn, G., Taylor, H., Morrison, A. P., and Lewis, S. W. (2013). Cognitive-self consciousness and metacognitive beliefs: Stress sensitization in individuals at ultra-high risk of developing psychosis. British Journal of Clinical Psychology, 52(1), 26–41.
[S261] Cristóbal-Narváez, P., Sheinbaum, T., Rosa, A., Ballespí, S., de Castro-Catala, M., Peña, E., … and Barrantes-Vidal, N. (2016). The interaction between childhood bullying and the FKBP5 gene on psychotic-like experiences and stress reactivity in real life. PLoS one, 11(7), e0158809.
[S262] Vani, M. F., Curran, T., and Sabiston, C. M. (2018). The relationship between physical activity and stress within women treated for breast cancer. Journal of Exercise, Movement, and Sport (SCAPPS refereed abstracts repository), 50(1), 312–312.
[S263] Gelkopf, M., Lapid Pickman, L., Carlson, E. B., and Greene, T. (2019). The dynamic relations among peritraumatic posttraumatic stress symptoms: An experience sampling study during wartime. Journal of traumatic stress, 32(1), 119–129.
[S264] Rauschenberg, C., Van Os, J., Cremers, D., Goedhart, M., Schieveld, J. N. M., and Reininghaus, U. (2017). Stress sensitivity as a putative mechanism linking childhood trauma and psychopathology in youth’s daily life. Acta Psychiatrica Scandinavica, 136(4), 373–388.
[S265] Booij, S. H., Snippe, E., Jeronimus, B. F., Wichers, M., and Wigman, J. T. (2018). Affective reactivity to daily life stress: Relationship to positive psychotic and depressive symptoms in a general population sample. Journal of affective disorders, 225, 474–481.
[S266] Poon, C. Y. S., Hui, V. K. Y., Yuen, G. W. C., Kwong, V. W. Y., and Chan, C. S. (2019). A well-slept teacher is a better teacher: A multi-respondent experience-sampling study on sleep, stress, and emotional transmission in the classroom. PsyCh journal, 8(3), 280–292.
[S267] Vork, L., Keszthelyi, D., van Kuijk, S. M., Quetglas, E. G., Törnblom, H., Simrén, M., … and Masclee, A. A. (2020). Patient-Specific Stress–Abdominal Pain Interaction in Irritable Bowel Syndrome: An Exploratory Experience Sampling Method Study. Clinical and Translational Gastroenterology, 11(7).
[S268] Wouters, S., Jacobs, N., Duif, M., Lechner, L., and Thewissen, V. (2018). Negative affective stress reactivity: The dampening effect of snacking. Stress and Health, 34(2), 286–295.
[S269] Tinajero, R., Williams, P. G., Cribbet, M. R., Rau, H. K., Silver, M. A., Bride, D. L., and Suchy, Y. (2020). Reported history of childhood trauma and stress-related vulnerability: Associations with emotion regulation, executive functioning, daily hassles and pre-sleep arousal. Stress and Health, 36(4), 405–418.
[S270] Klippel, A., Myin-Germeys, I., Chavez-Baldini, U., Preacher, K. J., Kempton, M., Valmaggia, L., … and Reininghaus, U. (2017). Modeling the interplay between psychological processes and adverse, stressful contexts and experiences in pathways to psychosis: an experience sampling study. Schizophrenia bulletin, 43(2), 302–315.
[S271] Rumbold, J., Fletcher, D., and Daniels, K. (2020). An experience sampling study of organizational stress processes and future playing time in professional sport. Journal of sports sciences, 38(5), 559–567.
[S272] Lapid Pickman, L., Greene, T., and Gelkopf, M. (2017). Sense of threat as a mediator of peritraumatic stress symptom development during wartime: An experience sampling study. Journal of Traumatic Stress, 30(4), 372–380.
[S273] Reininghaus, U., Gayer-Anderson, C., Valmaggia, L., Kempton, M. J., Calem, M., Onyejiaka, A., … and Morgan, C. (2016). Psychological processes underlying the association between childhood trauma and psychosis in daily life: an experience sampling study. Psychological medicine, 46(13), 2799–2813.
[S274] Brose, A., Wichers, M., and Kuppens, P. (2017). Daily stressful experiences precede but do not succeed depressive symptoms: Results from a longitudinal experience sampling study. Journal of Social and Clinical Psychology, 36(3), 196–220.
[S275] Myin-Germeys, I., Kasanova, Z., Vaessen, T., Vachon, H., Kirtley, O., Viechtbauer, W., and Reininghaus, U. (2018). Experience sampling methodology in mental health research: new insights and technical developments. World Psychiatry, 17(2), 123–132.
[S276] Udachina, A., Varese, F., Myin-Germeys, I., and Bentall, R. P. (2014). The role of experiential avoidance in paranoid delusions: an experience sampling study. British Journal of Clinical Psychology, 53(4), 422–432.
[S277] Pacella, M. L., Girard, J. M., Wright, A. G., Suffoletto, B., and Callaway, C. W. (2018). The association between daily posttraumatic stress symptoms and pain over the first 14 days after injury: An experience sampling study. Academic emergency medicine, 25(8), 844–855.
[S278] Ellison, W. D., Trahan, A. C., Pinzon, J. C., Gillespie, M. E., Simmons, L. M., and King, K. Y. (2020). For whom, and for what, is experience sampling more accurate than retrospective report?. Personality and Individual Differences, 163, 110071.
[S279] Block, V. J., Meyer, A. H., Miché, M., Mikoteit, T., Hoyer, J., Imboden, C., … and Gloster, A. T. (2020). The effect of anticipatory stress and openness and engagement on subsequently perceived sleep quality–An Experience Sampling Method study. Journal of sleep research, 29(5), e12957.
[S280] Shoham, A., Hadash, Y., and Bernstein, A. (2018). Examining the decoupling model of equanimity in mindfulness training: An intensive experience sampling study. Clinical Psychological Science, 6(5), 704–720.
[S281] Henquet, C., van Os, J., Kuepper, R., Delespaul, P., Smits, M., Campo, J. A., and Myin-Germeys, I. (2010). Psychosis reactivity to cannabis use in daily life: an experience sampling study. The British Journal of Psychiatry, 196(6), 447–453.
[S282] Kimhy, D., Wall, M. M., Hansen, M. C., Vakhrusheva, J., Choi, C. J., Delespaul, P., … and Malaspina, D. (2017). Autonomic regulation and auditory hallucinations in individuals with schizophrenia: an experience sampling study. Schizophrenia Bulletin, 43(4), 754–763.
[S283] van Os, J., Lataster, T., Delespaul, P., Wichers, M., and Myin-Germeys, I. (2014). Evidence that a psychopathology interactome has diagnostic value, predicting clinical needs: an experience sampling study. PLoS One, 9(1), e86652.
[S284] Keller, M. M., Chang, M. L., Becker, E. S., Goetz, T., and Frenzel, A. C. (2014). Teachers’ emotional experiences and exhaustion as predictors of emotional labor in the classroom: An experience sampling study. Frontiers in psychology, 5, 1442.
[S285] Walsh, K. M., Saab, B. J., and Farb, N. A. (2019). Effects of a mindfulness meditation app on subjective well-being: Active randomized controlled trial and experience sampling study. JMIR mental health, 6(1), e10844.
[S286] Peerbooms, O., Rutten, B. P. F., Collip, D., Lardinois, M., Lataster, T., Thewissen, V., … and van Winkel, R. (2012). Evidence that interactive effects of COMT and MTHFR moderate psychotic response to environmental stress. Acta Psychiatrica Scandinavica, 125(3), 247–256.
[S287] Serino, S., Cipresso, P., Tartarisco, G., Baldus, G., Corda, D., Pioggia, G., … and Riva, G. (2013). Computerized experience-sampling approach for realtime assessment of stress. EAI Endorsed Transactions on Ambient Systems, 1(2).
[S288] Lataster, T., Collip, D., Lardinois, M., Van Os, J., and Myin-Germeys, I. (2010). Evidence for a familial correlation between increased reactivity to stress and positive psychotic symptoms. Acta Psychiatrica Scandinavica, 122(5), 395–404.
[S289] Carstensen, L. L., Turan, B., Scheibe, S., Ram, N., Ersner-Hershfield, H., Samanez-Larkin, G. R., … and Nesselroade, J. R. (2011). Emotional experience improves with age: evidence based on over 10 years of experience sampling. Psychology and aging, 26(1), 21.
[S290] Poerio, G. L., Totterdell, P., Emerson, L. M., and Miles, E. (2016). Social daydreaming and adjustment: an experience-sampling study of socio-emotional adaptation during a life transition. Frontiers in psychology, 7, 13.
[S291] Kwapil, T. R., Brown, L. H., Silvia, P. J., Myin-Germeys, I., and Barrantes-Vidal, N. (2012). The expression of positive and negative schizotypy in daily life: an experience sampling study. Psychological Medicine, 42(12), 2555–2566.
[S292] Collip, D., Habets, P., Marcelis, M., Gronenschild, E., Lataster, T., Lardinois, M., … and Myin-Germeys, I. (2013). Hippocampal volume as marker of daily life stress sensitivity in psychosis. Psychological Medicine, 43(7), 1377–1387.
[S293] Habets, P., Collip, D., Myin-Germeys, I., Gronenschild, E., Van Bronswijk, S., Hofman, P., … and Marcelis, M. (2012). Pituitary volume, stress reactivity and genetic risk for psychotic disorder. Psychological medicine, 42(7), 1523–1533.
[S294] Collip, D., Wigman, J. T., Myin-Germeys, I., Jacobs, N., Derom, C., Thiery, E., … and van Os, J. (2013). From epidemiology to daily life: linking daily life stress reactivity to persistence of psychotic experiences in a longitudinal general population study. PloS one, 8(4), e62688.
[S295] Wijngaards, I., Hendriks, M., and Burger, M. J. (2019). Steering towards happiness: An experience sampling study on the determinants of happiness of truck drivers. Transportation research part A: policy and practice, 128, 131–148.
[S296] Riedl, E. M., and Thomas, J. (2019). The moderating role of work pressure on the relationships between emotional demands and tension, exhaustion, and work engagement: an experience sampling study among nurses. European Journal of Work and Organizational Psychology, 28(3), 414–429.
[S297] Rintala, A., Wampers, M., Myin-Germeys, I., and Viechtbauer, W. (2019). Response compliance and predictors thereof in studies using the experience sampling method. Psychological assessment, 31(2), 226.
[S298] Moeller, J., Brackett, M. A., Ivcevic, Z., and White, A. E. (2020). High school students’ feelings: Discoveries from a large national survey and an experience sampling study. Learning and Instruction, 66, 101301.
[S299] Lüdtke, T., Kriston, L., Schröder, J., Lincoln, T. M., and Moritz, S. (2017). Negative affect and a fluctuating jumping to conclusions bias predict subsequent paranoia in daily life: an online experience sampling study. Journal of Behavior Therapy and Experimental Psychiatry, 56, 106–112.
[S300] Fitzgerald-DeJean, D. M., Rubin, S. S., and Carson, R. L. (2012). An application of the experience sampling method to the study of aphasia: A case report. Aphasiology, 26(2), 234–251.
[S301] Verhagen, S. J., Berben, J. A., Leue, C., Marsman, A., Delespaul, P. A., van Os, J., and Lousberg, R. (2017). Demonstrating the reliability of transdiagnostic mHealth Routine Outcome Monitoring in mental health services using experience sampling technology. PloS one, 12(10), e0186294.
[S302] Brown, L. H., Strauman, T., Barrantes-Vidal, N., Silvia, P. J., and Kwapil, T. R. (2011). An experience-sampling study of depressive symptoms and their social context. The Journal of nervous and mental disease, 199(6), 403–409.
[S303] Uy, M. A., Foo, M. D., and Aguinis, H. (2010). Using experience sampling methodology to advance entrepreneurship theory and research. Organizational Research Methods, 13(1), 31–54.
[S304] Palmier-Claus, J. E., Dunn, G., Taylor, H., Morrison, A. P., and Lewis, S. W. (2013). Cognitive-self consciousness and metacognitive beliefs: Stress sensitization in individuals at ultra-high risk of developing psychosis. British Journal of Clinical Psychology, 52(1), 26–41.
[S305] Palmier-Claus, J. E., Taylor, P. J., Gooding, P., Dunn, G., and Lewis, S. W. (2012). Affective variability predicts suicidal ideation in individuals at ultra-high risk of developing psychosis: An experience sampling study. British Journal of Clinical Psychology, 51(1), 72–83.
[S306] Elhai, J. D., Rozgonjuk, D., Liu, T., and Yang, H. (2020). Fear of missing out predicts repeated measurements of greater negative affect using experience sampling methodology. Journal of affective disorders, 262, 298–303.
[S307] Geschwind, N., Peeters, F., Jacobs, N., Delespaul, P., Derom, C., Thiery, E., … and Wichers, M. (2010). Meeting risk with resilience: high daily life reward experience preserves mental health. Acta Psychiatrica Scandinavica, 122(2), 129–138.
[S308] Hurlburt, R. T., and Heavey, C. L. (2015). Investigating pristine inner experience: implications for experience sampling and questionnaires. Consciousness and Cognition, 31, 148–159.
[S309] Kent, B. V., Henderson, W. M., Bradshaw, M., Ellison, C. G., and Wright, B. R. (2021). Do Daily Spiritual Experiences Moderate the Effect of Stressors on Psychological Well-being? A Smartphone-based Experience Sampling Study of Depressive Symptoms and Flourishing. The International Journal for the Psychology of Religion, 31(2), 57–78.
[S310] Simor, P., Báthori, N., Nagy, T., and Polner, B. (2019). Poor sleep quality predicts psychotic-like symptoms: an experience sampling study in young adults with schizotypal traits. Acta Psychiatrica Scandinavica, 140(2), 135–146.
[S311] Nielsen, K., and Cleal, B. (2011). Under which conditions do middle managers exhibit transformational leadership behaviors?—An experience sampling method study on the predictors of transformational leadership behaviors. The Leadership Quarterly, 22(2), 344–352.
[S312] Hartmann, J. A., Wichers, M., Menne-Lothmann, C., Kramer, I., Viechtbauer, W., Peeters, F., … and Simons, C. J. (2015). Experience sampling-based personalized feedback and positive affect: a randomized controlled trial in depressed patients. PLoS One, 10(6), e0128095.
[S313] Becker, E. S., Goetz, T., Morger, V., and Ranellucci, J. (2014). The importance of teachers’ emotions and instructional behavior for their students’ emotions–An experience sampling analysis. Teaching and Teacher Education, 43, 15–26.
[S314] Kim, D., Lam, J., Kutz, A., and Yoon, K. L. (2021). Punishment sensitivity and risk taking in depressed mood. Motivation and Emotion, 45(1), 122–130.
[S315] Ghosh, S., Mandi, S., Mitra, B., and De, P. (2021, April). Exploring Smartphone Keyboard Interactions for Experience Sampling Method driven Probe Generation. In 26th International Conference on Intelligent User Interfaces (pp. 133–138).
[S316] Lishinski, A., Rosenberg, J., Mann, M., Sultana, O., and Dunn, J. (2021, March). How CS1 Students Experienced COVID-19 In the Moment: Using An Experience Sampling Approach to Understand the Transition to Emergency Remote Instruction. In Proceedings of the 52nd ACM Technical Symposium on Computer Science Education (pp. 1254–1254).
[S317] Choi, H., Lee, W., and Hyland, P. (2021). Factor structure and symptom classes of ICD-11 complex posttraumatic stress disorder in a South Korean general population sample with adverse childhood experiences. Child Abuse and Neglect, 114, 104982.
[S318] Sladek, M. R., Castro, S. A., and Doane, L. D. (2021). Ethnic-Racial discrimination experiences predict Latinx adolescents’ physiological stress processes across college transition. Psychoneuroendocrinology, 128, 105212.
[S319] Fiore, J. (2021). Randomized pilot study exploring an online pre-composed receptive music experience and a mindfulness-based intervention for hospice workers’ stress and professional quality of life. The Arts in Psychotherapy, 74, 101797.
[S320] Rauschenberg, C., van Os, J., Goedhart, M., Schieveld, J. N., and Reininghaus, U. (2021). Bullying victimization and stress sensitivity in help-seeking youth: findings from an experience sampling study. European child and adolescent psychiatry, 30(4), 591.
[S321] Panlilio, L. V., Stull, S. W., Bertz, J. W., Burgess-Hull, A. J., Lanza, S. T., Curtis, B. L., … and Preston, K. L. (2021). Beyond abstinence and relapse II: momentary relationships between stress, craving, and lapse within clusters of patients with similar patterns of drug use. Psychopharmacology, 1–17.
[S322] Pätzold, I., Myin-Germeys, I., Schick, A., Nelson, B., Velthorst, E., Schirmbeck, F., … and Reininghaus, U. (2021). Stress reactivity as a putative mechanism linking childhood trauma with clinical outcomes in individuals at ultra-high-risk for psychosis: Findings from the EU-GEI High Risk Study. Epidemiology and Psychiatric Sciences, 30.
[S323] Soto, M., Satterfield, C., Fritz, T., Murphy, G. C., Shepherd, D. C., and Kraft, N. (2021). Observing and predicting knowledge worker stress, focus and awakeness in the wild. International Journal of Human-Computer Studies, 146, 102560.
[S324] Zareian, B., Wilson, J., and LeMoult, J. (2021). Cognitive control and ruminative responses to stress: Understanding the different facets of cognitive control. Frontiers in Psychology, 12, 1594.
[S325] Klippel, A., Schick, A., Myin-Germeys, I., Rauschenberg, C., Vaessen, T., and Reininghaus, U. (2021). Modelling the temporal interplay between stress and affective disturbances in pathways to psychosis: an experience sampling study. Psychological Medicine, 1–10.
[S326] Akbar, F., Mark, G., Prausnitz, S., Warton, E. M., East, J. A., Moeller, M. F., … and Lieu, T. A. (2021). Physician Stress During Electronic Health Record Inbox Work: In Situ Measurement With Wearable Sensors. JMIR Medical Informatics, 9(4), e24014.
[S327] Määttänen, I., Henttonen, P., Väliaho, J., Palomäki, J., Thibault, M., Kallio, J., … and Jokela, M. (2021). Positive affect state is a good predictor of movement and stress: combining data from ESM/EMA, mobile HRV measurements and trait questionnaires. Heliyon, 7(2), e06243.
[S328] Wolff, M., Enge, S., Kräplin, A., Krönke, K. M., Bühringer, G., Smolka, M. N., and Goschke, T. (2021). Chronic stress, executive functioning, and real-life self-control: An experience sampling study. Journal of Personality, 89(3), 402–421.
[S329] von Harling, H. (2021). Are you satisfied?: The role of basic psychological need satisfaction in the perception of stress in university students: an experience sampling study (Bachelor’s thesis, University of Twente).
[S330] Shigemoto, Y. (2021). Association between daily rumination and posttraumatic growth during the COVID-19 pandemic: An experience sampling method. Psychological Trauma: Theory, Research, Practice, and Policy.
[S331] Pejovic, V., Lathia, N., Mascolo, C., and Musolesi, M. (2016). Mobile-based experience sampling for behaviour research. In Emotions and personality in personalized services (pp. 141–161). Springer, Cham.
[S332] Engström, A., and Kronkvist, K. (2018). Situating fear of crime: The prospects for criminological research to use smartphone applications to gather experience sampling data. In Community-oriented policing and technological innovations (pp. 85–93). Springer, Cham.
[S333] Petrie, H., Carmien, S., and Lewis, A. (2017, July). Obtaining Experiential Data on Assistive Technology Device Abandonment. In International Conference on Universal Access in Human-Computer Interaction (pp. 217–226). Springer, Cham.
[S334] Kappelgaard, L. H., and Lund, K. (2013, July). Ecological momentary storytelling: Bringing down organizational stress through qualifying work life stories. In International Conference on Augmented Cognition (pp. 572–581). Springer, Berlin, Heidelberg.
[S335] Mendes, M. S., Furtado, E., Furtado, V., and de Castro, M. F. (2014, June). How do users express their emotions regarding the social system in use? A classification of their postings by using the emotional analysis of Norman. In International Conference on Social Computing and Social Media (pp. 229–241). Springer, Cham.
[S336] Tschacher, W., and Lienhard, N. (2021). Mindfulness is linked with affectivity in daily life: An experience-sampling study with meditators. Mindfulness, 12(6), 1459–1472. Chicago
[S337] Bringmann, L. F., van der Veen, D. C., Wichers, M., Riese, H., and Stulp, G. (2021). ESMvis: a tool for visualizing individual Experience Sampling Method (ESM) data. Quality of Life Research, 30(11), 3179–3188.
[S338] Stieger, S., Lewetz, D., and Swami, V. (2021). Emotional well-being under conditions of lockdown: An experience sampling study in Austria during the COVID-19 pandemic. Journal of happiness studies, 22(6), 2703–2720.
[S339] Kärner, T., and Höning, J. (2021). Teachers’ experienced classroom demands and autonomic stress reactions: results of a pilot study and implications for process-oriented research in vocational education and training. Empirical Research in Vocational Education and Training, 13(1), 1–22.
[S340] Li, X., Zhang, Y., and Huang, J. (2021, September). Testing a design-based learning approach to enhance elementary students’ computational thinking with experience-sampling method. In 2021 The 3rd World Symposium on Software Engineering (pp. 17–22).
[S341] Paananen, V., Oppenlaender, J., Goncalves, J., Hettiachchi, D., and Hosio, S. (2021). Investigating Human Scale Spatial Experience. Proceedings of the ACM on Human-Computer Interaction, 5(ISS), 1–18.
[S342] Lyu, M., Zhang, Z., and Sun, B. (2021, August). How Does the Use of Mobile Social Platforms Impact Impulsive Buying? A Case of Wechat. In 2021 4th International Conference on Information Management and Management Science (pp. 31–35).
[S343] Pickman, L. L., Gelkopf, M., and Greene, T. (2021). Do positive and negative emotional reactions during war predict subsequent symptomatology? A prospective experience sampling study. Journal of Anxiety Disorders, 84, 102492.
[S344] Eisele, G., Lafit, G., Vachon, H., Kuppens, P., Houben, M., Myin-Germeys, I., and Viechtbauer, W. (2021). Affective structure, measurement invariance, and reliability across different experience sampling protocols. Journal of Research in Personality, 92, 104094.
[S345] Gadosey, C. K., Schnettler, T., Scheunemann, A., Fries, S., and Grunschel, C. (2021). The intraindividual co-occurrence of anxiety and hope in procrastination episodes during exam preparations: An experience sampling study. Learning and Individual Differences, 88, 102013.
[S346] De Vries, S., Nieuwenhuizen, W., Farjon, H., Van Hinsberg, A., and Dirkx, J. (2021). In which natural environments are people happiest? Large-scale experience sampling in the Netherlands. Landscape and urban planning, 205, 103972.
[S347] Leenaerts, N., Vaessen, T., Ceccarini, J., and Vrieze, E. (2021). How COVID-19 lockdown measures could impact patients with bulimia nervosa: Exploratory results from an ongoing experience sampling method study. Eating behaviors, 41, 101505.
[S348] Hallard, R. I., Wells, A., Aadahl, V., Emsley, R., and Pratt, D. (2021). Metacognition, rumination and suicidal ideation: An experience sampling test of the self-regulatory executive function model. Psychiatry Research, 303, 114083.
[S349] Dietvorst, E., Hiemstra, M., Maciejewski, D., van Roekel, E., Ter Bogt, T., Hillegers, M., and Keijsers, L. (2021). Grumpy or depressed? Disentangling typically developing adolescent mood from prodromal depression using experience sampling methods. Journal of adolescence, 88, 25–35.
[S350] Janssens, M., Janssens, E., Eshuis, J., Lataster, J., Simons, M., Reijnders, J., and Jacobs, N. (2021). Companion Animals as Buffer against the Impact of Stress on Affect: An Experience Sampling Study. Animals, 11(8), 2171.
[S351] Dančík, D., Kasanova, Z., Hajdúk, M., and Heretik, A. (2021). Attachment, Stress and Emotions in Daily Life: An Experience Sampling Study. Studia Psychologica, 63(4), 323–336.
[S352] Cipresso, P., Serino, S., Borghesi, F., Tartarisco, G., Riva, G., Pioggia, G., and Gaggioli, A. (2021). Continuous measurement of stress levels in naturalistic settings using heart rate variability: An experience-sampling study driving a machine learning approach.
[S353] Lapid Pickman, L., Gelkopf, M., and Greene, T. (2021). Emotional reactivity to war stressors: An experience sampling study in people with and without different psychiatric diagnoses. Stress and Health, 37(1), 127–139.
[S354] Klaassen, T., Vork, L., Smeets, F. G., Troost, F. J., Kruimel, J. W., Leue, C., … and Keszthelyi, D. (2021). The interplay between stress and fullness in functional dyspepsia and healthy controls: an exploratory experience sampling method study. Novel strategies to address disrupted sensing and signalling of satiety.
[S355] Xu, S., Li, W., Zhang, W., and Cho, J. (2021). The dynamics of social support and affective well-being before and during COVID: An experience sampling study. Computers in Human Behavior, 121, 106776.
[S356] Daily Work-Family Conflict and Burnout to Explain the Leaving Intentions and Vitality Levels of Healthcare Workers: Interactive Effects Using an Experience-Sampling Method
[S357] Johannes, N., Meier, A., Reinecke, L., Ehlert, S., Setiawan, D. N., Walasek, N., … and Veling, H. (2021). The relationship between online vigilance and affective well-being in everyday life: Combining smartphone logging with experience sampling. Media Psychology, 24(5), 581–605.
[S358] Zhou, B., Li, Y., Tang, Y., and Cao, W. (2021). An experience-sampling study on academic stressors and cyberloafing in college students: The moderating role of trait self-control. Frontiers in Psychology, 12.
